# Distinct roles of COPI proteins attenuated in cell senescence

**DOI:** 10.1126/sciadv.aec2786

**Published:** 2026-07-23

**Authors:** Krystyna Mazan-Mamczarz, Eleanor J. Wind, Apala Pal, Martin Salamini-Montemurri, Dimitrios Tsitsipatis, Kyoung Mi Kim, Rachel Munk, Jixiang Leng, Chang Hoon Shin, Jennifer L. Martindale, Qiong Meng, Martina Rossi, Yulan Piao, Marika Oksanen, Sarah Buchman, Braden Daugherty, Jinshui Fan, Supriyo De, Allison B. Herman, Myriam Gorospe

**Affiliations:** ^1^ Laboratory of Genetics and Genomics (LGG), National Institute on Aging Intramural Research Program (NIA IRP), National Institutes of Health (NIH), Baltimore, MD 21224, USA.; ^2^ Laboratory of Cardiovascular Science (LCS), National Institute on Aging Intramural Research Program (NIA IRP), National Institutes of Health (NIH), Baltimore, MD 21224, USA.; ^3^ Department of Biological Sciences, Chungnam National University, Daejeon 34134, Republic of Korea.

## Abstract

In senescent cells, functional alterations in organelles like mitochondria and lysosomes are well characterized, but senescence-associated changes in Golgi function are not. An RNA interference screen revealed that silencing subunits of the coatomer protein I (COPI) complex, critical for intracellular transport involving the Golgi, reduced extracellular vesicle uptake. In proliferating WI-38 fibroblasts, silencing COPI constituents COPA, COPB1, COPB2, or COPD induced ATF4 production and disrupted autophagy, apoptosis, and cytokine signaling, all hallmarks of impaired Golgi–to–endoplasmic reticulum transport, while silencing COPI constituents COPG1, COPE, or COPZ1 altered the production of extracellular matrix proteins. Furthermore, individual COPI proteins associated with Golgi and endosomal proteins, supporting roles in vesicular trafficking. In senescent WI-38 fibroblasts, silencing COPI proteins did not elicit these phenotypes. Our findings underscore the distinct, multifunctional actions of individual COPI proteins and their key roles in intracellular homeostatic transport networks that become attenuated during senescence.

## Teaser

Individual COPI protein components have distinct functions in dividing cells, and COPI function is attenuated in senescent cells.

## INTRODUCTION

Cellular senescence is a state of indefinite growth arrest triggered in response to sublethal damage. Senescent cells have increased resistance to apoptosis, undergo changes in their transcriptomes and proteomes, and exhibit a senescence-associated secretory phenotype (SASP). In addition, senescent cells display alterations in the structure, size, and function of various organelles, which change cell homeostasis and interactions with neighboring cells (*1*, *2*). For example, mitochondria in senescent cells produce more reactive oxygen species (*3*), and lysosomes display increased β-galactosidase activity (*1*, *2*). Previous research has also reported a distorted Golgi structure in senescent cells (*4*–*6*) linked to age-related diseases (*7*, *8*), but the impact of Golgi function on the senescence program is poorly understood.

The Golgi apparatus is a major organelle that governs the processing of proteins, lipids, and carbohydrates and coordinates the formation and trafficking of vesicles to intracellular sites (*9*). In particular, proteins in the coatomer protein complex I (COPI) system play a key role in forming a coat around budding Golgi vesicles, primarily on the cis-Golgi [near the endoplasmic reticulum (ER) side], facilitating the retrieval of escaped ER proteins and recycling of Golgi enzymes back to the ER, a process known as retrograde transport (*10*). COPI proteins also mediate the lesser-understood anterograde transport of vesicles from the ER to Golgi and transport between the Golgi and the endosomal system (*11*, *12*). By mediating vesicle formation and delivery, the COPI system helps preserve the dynamic function and organization of the Golgi apparatus within intracellular communication routes. The COPI complex consists of seven subunits: COPA (αCOP), COPB1 (βCOP), COPB2 (β′COP), COPD (δCOP, ARCN1), COPE (εCOP), COPG1 (γCOP), and COPZ1 (ξCOP), which are stably associated and recruited to the membrane to facilitate vesicle formation, cargo selection, and transport (*10*). Deficiencies in specific COPI subunits led to immune dysregulation (*13*–*16*) and reduced susceptibility to some infections (*17*, *18*).

Through an RNA interference (RNAi) screen to identify proteins implicated in the uptake of extracellular vesicles (EVs), we found that silencing protein components of COPI diminished EV uptake by human cervical carcinoma HeLa cells and human diploid WI-38 fibroblasts. The further findings that senescent WI-38 fibroblasts displayed diminished EV uptake and reduced expression of COPI coatomer proteins prompted us to evaluate the function of COPI subunits in WI-38 fibroblasts that were either proliferating [nonsenescent (NS)] or had undergone etoposide-induced senescence (ETIS). Individually silencing each COPI protein in proliferating WI-38 NS cells influenced distinct cellular pathways: Silencing COPA, COPB1, COPB2, or COPD induced ER stress and activating transcription factor 4 (ATF4) signaling, while silencing COPG1, COPZ1, or COPE changed the production of extracellular matrix (ECM) components. Reducing any of the COPI proteins lowered mitochondrial respiration and, with the exception of COPE, decreased the expression of cell proliferation factors.

Senescent cells were not affected by further decreases in COPI protein levels, underscoring their reduced ability to mount stress responses. Immunoprecipitation-mass spectrometry (IP-MS) analysis identified an association of COPI complex proteins with components of the Golgi membrane and the endosomal networks, connecting COPI to the vesicular trafficking between these compartments. In summary, we report distinct, multifunctional traits of individual proteins coating COPI vesicles and propose that they contribute to the dynamic intracellular transport networks critical for maintaining cell homeostasis and adaptive responses, which become impaired in senescent cells.

## RESULTS

### RNAi screen identified COPI vesicle coat proteins as required for EV uptake

**Fig. 1.**
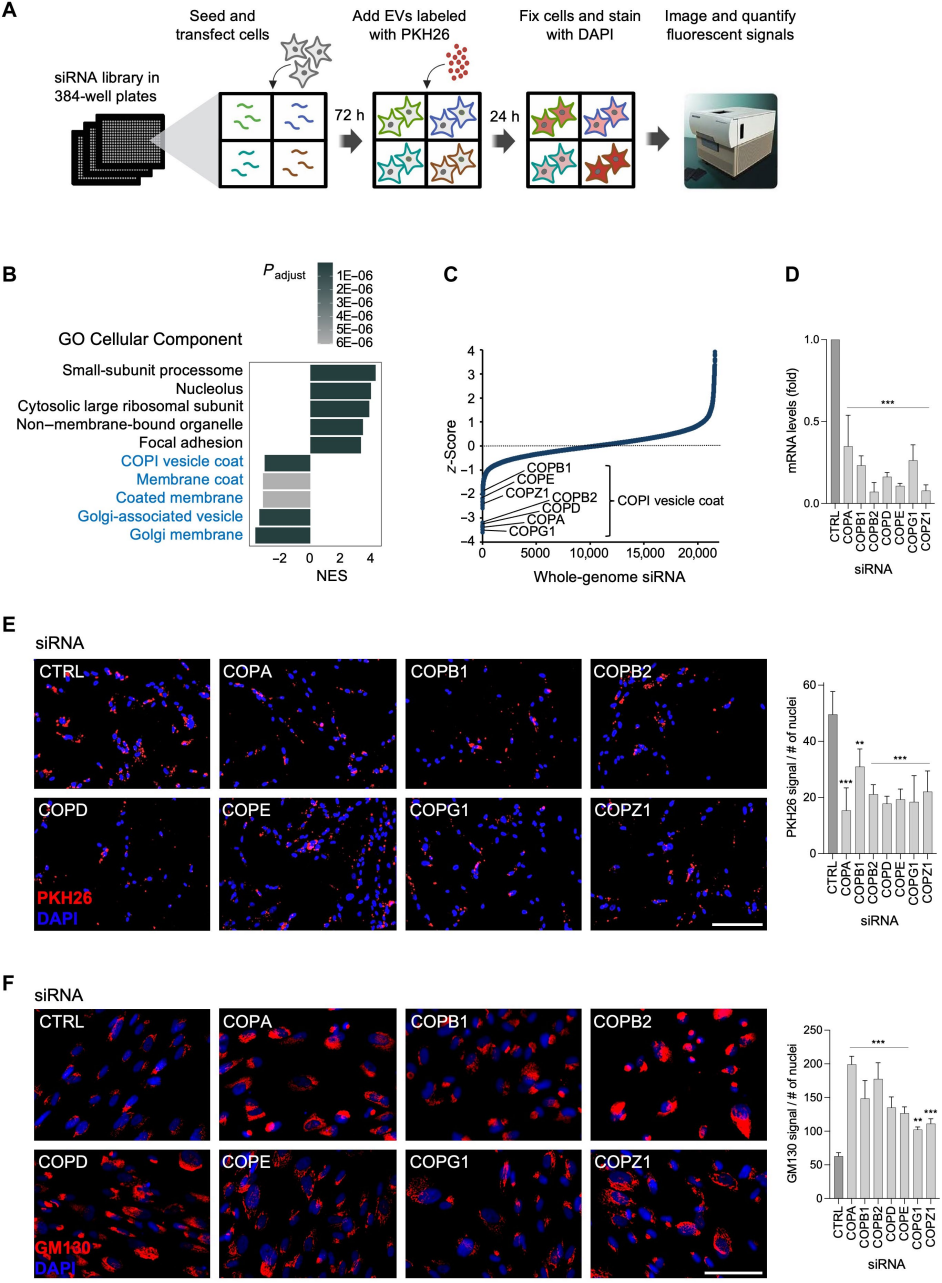
Identification of subunits of COPI vesicle coat as modifiers of EV uptake in genome-wide RNAi screen. (**A**) Workflow of RNAi screen to identify regulators of EV uptake, including reverse transfection, incubation with PKH26-labeled EVs, and identification of cells exhibiting altered label uptake. h, hours. (**B**) GSEA of GO CC terms performed on the top genes identified by RNAi screen whose silencing altered the uptake of EVs (*z*-score cutoff >1 and <−1). NES, normalized enrichment score for activated and suppressed terms. (**C**) Ranked distribution of median seed-corrected *z*-score for individual siRNAs in the RNAi screen of EV uptake. Protein components of the COPI vesicle coat complex are indicated. (**D**) WI-38 fibroblasts were transfected with either nontargeting siRNA (CTRL) or siRNAs designed to silence the different subunits of the COPI complex; 72 hours later, the transfection efficiency in each group was measured by RT-qPCR analysis. Relative mRNA levels were normalized to *GAPDH* mRNA levels in each transfection group, and all were compared to the levels in CTRL siRNA cells (*n* = 3). (**E**) Representative fluorescent micrographs (left) and quantification (*n* = 4) (right) of the uptake of EVs labeled with PKH26 dye (red) by WI-38 cells after silencing each component of the COPI complex. Nuclei (blue) were labeled with DAPI. Scale bar, 200 μm. (**F**) Representative micrographs (left) and quantification (*n* = 5) (right) of changes in the Golgi compartment after silencing each COPI component, as visualized by immunofluorescence microscopy using an antibody that recognizes the Golgi membrane protein GM130 (red); nuclei (blue) were labeled with DAPI. Scale bar, 100 μm. In (D) to (F), statistical significance was assessed by ANOVA followed by Dunnett’s post hoc test (*P < 0.05; **P < 0.01; ***P < 0.001) compared to CTRL siRNA.

In an earlier investigation, we sought to identify modulators of EV uptake from the surrounding environment by carrying out an arrayed, image-based genome-wide RNAi screen in human cervical carcinoma HeLa cells (Fig. 1A). Briefly, cells were reverse transfected in 384-well plates printed with an individual small interfering RNA (siRNA) in each well; 72 hours later, the transfected cells were cultured with EV particles labeled with the fluorescent dye PKH26, and an additional 24 hours later, fluorescent images of siRNA-transfected cells were quantitatively analyzed by automated microscopy. The fluorescent signals indicating the uptake of EV by the cells were then converted to *z*-scores and ranked to select candidates that promoted or reduced EV uptake by the cell (Materials and Methods and table S1).

Functional Gene Set Enrichment Analysis (GSEA) using Gene Ontology (GO) terms revealed that the top gene products whose knockdown impeded the uptake of EVs (*z*-score < −1) encoded components of the Golgi membrane vesicle network and COPI vesicle coat system (Fig. 1B). As shown, the COPI subunits COPA, COPB1, COPB2, COPD, COPE, COPG1, and COPZ1 were found among those with the lowest *z*-score on the RNAi screen, indicating that silencing the respective mRNAs robustly decreased EV uptake (Fig. 1C). Validation of the RNAi screen was carried out using an independent set of siRNAs (Dharmacon, Inc.; table S2) transfected into WI-38 cells and analyzed 72 hours later. The efficiency of the silencing was evaluated by collecting total RNA and measuring the levels of each COPI mRNA by reverse transcription (RT) followed by real-time quantitative polymerase chain reaction (qPCR) analysis (Fig. 1D). The reduced efficiency of the uptake of PKH26-labeled EVs by WI-38 cells after silencing COPI coat proteins was confirmed by fluorescence microscopy (Fig. 1E, left). We observed significant reductions in EV uptake (>50%) after silencing each COPI subunit compared to the control group (CTRL siRNA; Fig. 1E, right). Given that the COPI coat proteins primarily reside in the Golgi membrane and are involved in maintaining the Golgi structure and transporting cargo between cisternae (*9*, *19*), we investigated whether the reduction of each COPI subunit influenced the Golgi compartment in WI-38 cells. Analysis of the Golgi membrane marker GM130 by immunofluorescence microscopy revealed that silencing individual COPI subunits markedly increased the Golgi compartment (Fig. 1F), supporting the notion that COPI proteins affect Golgi organization and more broadly influence intracellular trafficking. Together, these results indicate that alterations in the expression of the COPI coat protein complex affect EV uptake and the size of the Golgi, suggesting a broader involvement of COPI proteins in vesicular transport in cells.

### Senescent cells exhibit altered Golgi compartment, diminished EV uptake, and reduced expression of COPI protein components

**Fig. 2.**
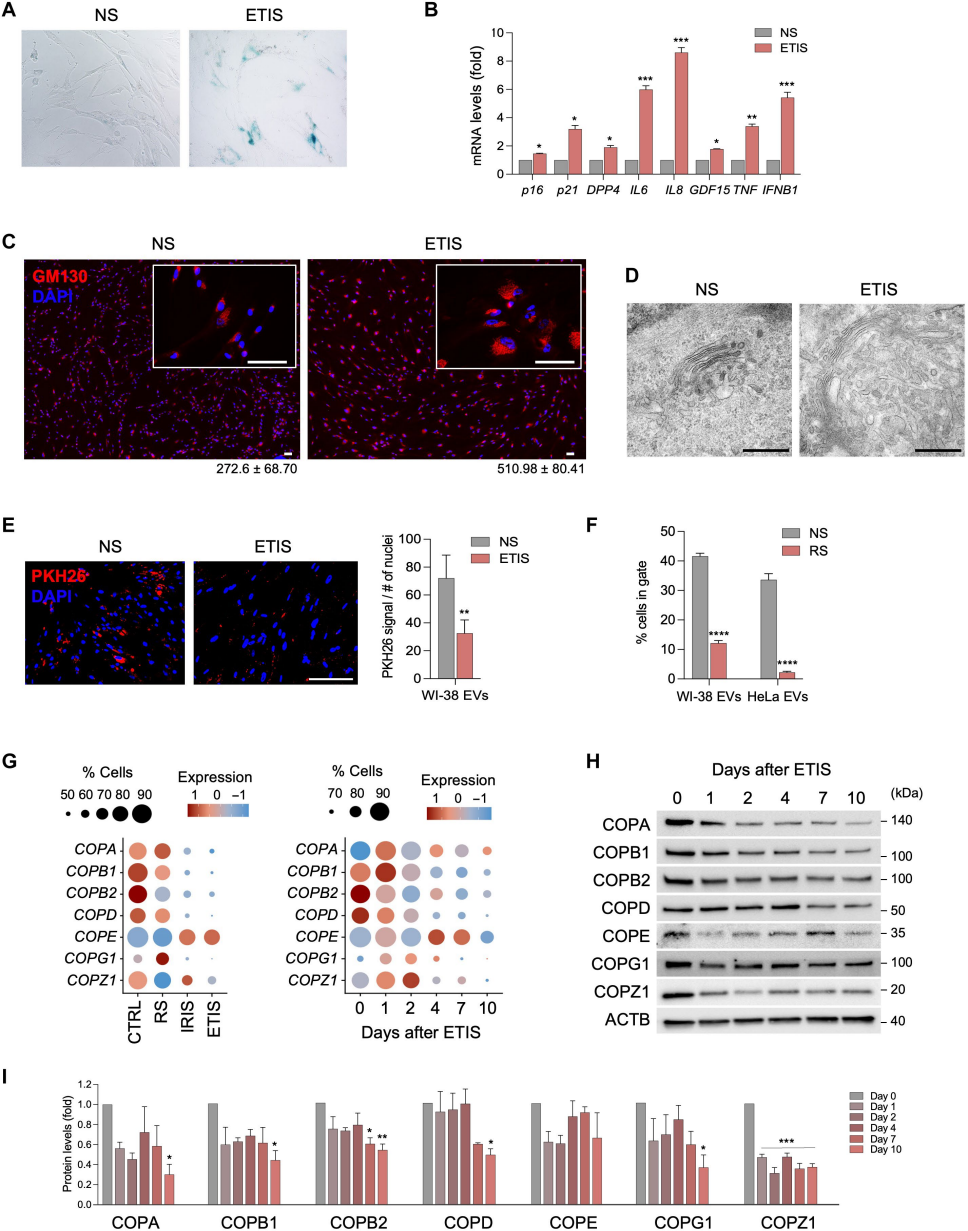
Altered Golgi compartment, uptake of EVs, and expression of COPI subunits in senescent cells. WI-38 fibroblasts were treated with 50 μM etoposide for 10 days to trigger ETIS or with DMSO (NS cells), whereupon they were analyzed. (**A**) Representative micrographs visualizing SA-β-Gal activity (blue) in NS and ETIS cells. (**B**) RT-qPCR analysis of the levels of senescence marker mRNAs in NS (gray) and ETIS (red) cells (*n* = 3). Data were normalized to *GAPDH* mRNA levels and represented as fold changes relative to NS. (**C**) Golgi compartment, visualized by staining the Golgi membrane protein GM130 (red) under fluorescence microscopy. Nuclei were stained with DAPI (blue). Scale bars, 100 μm. GM130-positive area per cell is indicated (*n* = 4). (**D**) Structure of the Golgi compartment in NS and ETIS cells (TEM). Scale bars, 500 nm. (**E**) Fluorescence micrograph visualization (left) and quantification (right) of the uptake of PKH26-labeled EVs (red); blue, DAPI-stained nuclei (*n* = 6). Scale bar, 200 μm. (**F**) Uptake of PKH26-labeled EVs by NS and RS WI-38 cells was quantified by flow cytometry (*n* = 3). (**G**) Abundance of mRNAs encoding COPI subunits in different senescence paradigms (left) and different times after triggering ETIS (right) in scRNA-seq datasets (*22*). Color, average expression; dot size, percentage of cells expressing specific mRNAs. CTRL, NS cells. (**H**) Western blot analysis of the levels of each COPI component after triggering ETIS. ACTB (β-actin), loading control. (**I**) Quantification of COPI signals from (H) (*n* = 3), normalized to ACTB levels and compared to day 0 (NS cells). Statistical significance was determined using an ANOVA followed by Dunnett’s post hoc test compared to day 0. In (B), (E), and (F), statistical significance was assessed by Student’s *t* test relative to NS cells. *P < 0.05; **P < 0.01; ***P < 0.001.

Aberrancies in Golgi function and impaired intracellular vesicular trafficking have been observed in senescent cells (*4*, *5*, *20*). We thus sought to study the levels of COPI proteins and the uptake of EVs in senescent cells. We used a previously established model of ETIS in human diploid WI-38 fibroblasts (*21*). Briefly, senescence was triggered by culturing cells in media containing 50 μM etoposide for 10 days, which causes sublethal damage leading to the cessation of cell proliferation (*21*). Cell senescence was confirmed by the rise in senescence-associated β-galactosidase (SA-β-Gal) activity (Fig. 2A) and the increased levels of mRNAs encoding senescence markers p16 (*CDKN2A* mRNA), p21 (*CDKN1A* mRNA), and DPP4 (*DPP4* mRNA), as well as mRNAs encoding SASP factors (*IL8*, *IL6*, *GDF15*, *TNF*, and *IFNB1* mRNAs; Fig. 2B). The Golgi compartment in the cell was examined by immunofluorescence staining of the Golgi network marker GM130 (Fig. 2C) and by using transmission electron microscopy (TEM) (Fig. 2D). In line with earlier reports (*4*, *5*, *20*), senescent cells displayed an enlarged and dispersed Golgi when compared to the small and compact Golgi seen in NS WI-38 cells. These changes in Golgi morphology were also observed in other senescence paradigms, including WI-38 fibroblasts in ionizing radiation–induced senescence (IRIS; fig. S1A) and human vascular smooth muscle cells (hVSMCs) in ETIS (fig. S1B).

To assess whether senescent cells exhibit an altered ability to uptake EVs, we cultured WI-38 fibroblasts with PKH26-stained EVs derived from HeLa cells; 24 hours later, the uptake of EVs by ETIS cells was reduced relative to the uptake by NS cells (Fig. 2E). We also found reduced uptake of PKH26-labeled EVs (derived from either HeLa or WI-38 cells) by WI-38 fibroblasts that had reached replicative senescence (RS) following extended culture through replicative exhaustion, as reported previously (Fig. 2F) (*22*).

To examine the abundance of COPI subunits in senescence, we analyzed single-cell RNA sequencing (scRNA-seq) datasets in different WI-38 cell senescence models, including ETIS, RS, and IRIS (*22*). This analysis revealed that the levels of mRNAs encoding the different COPI members generally declined in all models of senescence, except *COPE* mRNA, which showed either no difference or increased levels (Fig. 2G). We also found that the reduction in mRNAs encoding individual COPI subunits was different for each senescence model and that, within each senescent population, there were subpopulations (clusters 0 to 7) of cells displaying different degrees of reduction (fig. S2A). Western blot analysis of the changes in each protein component of COPI over time after triggering ETIS revealed decreases in the abundance of several proteins with the exception of COPE, even though the magnitude and rate of reduction of the protein levels differed across the various COPI components (Fig. 2, H and I); a schematic of the COPI coatomer complex components is in fig. S2B.

These data suggest that both the aberrant Golgi structure and lower levels of various COPI subunits in senescent cells may be implicated in the impairment of COPI vesicle function, in turn affecting intracellular vesicular transport and EV uptake. Furthermore, the varying levels of COPI components in different senescence paradigms, along with their inconsistent changes during senescence, suggest that individual COPI subunits may have diverse functions in cells.

### RNA-seq data reveal functional differences among subunits of the COPI complex

**Fig. 3.**
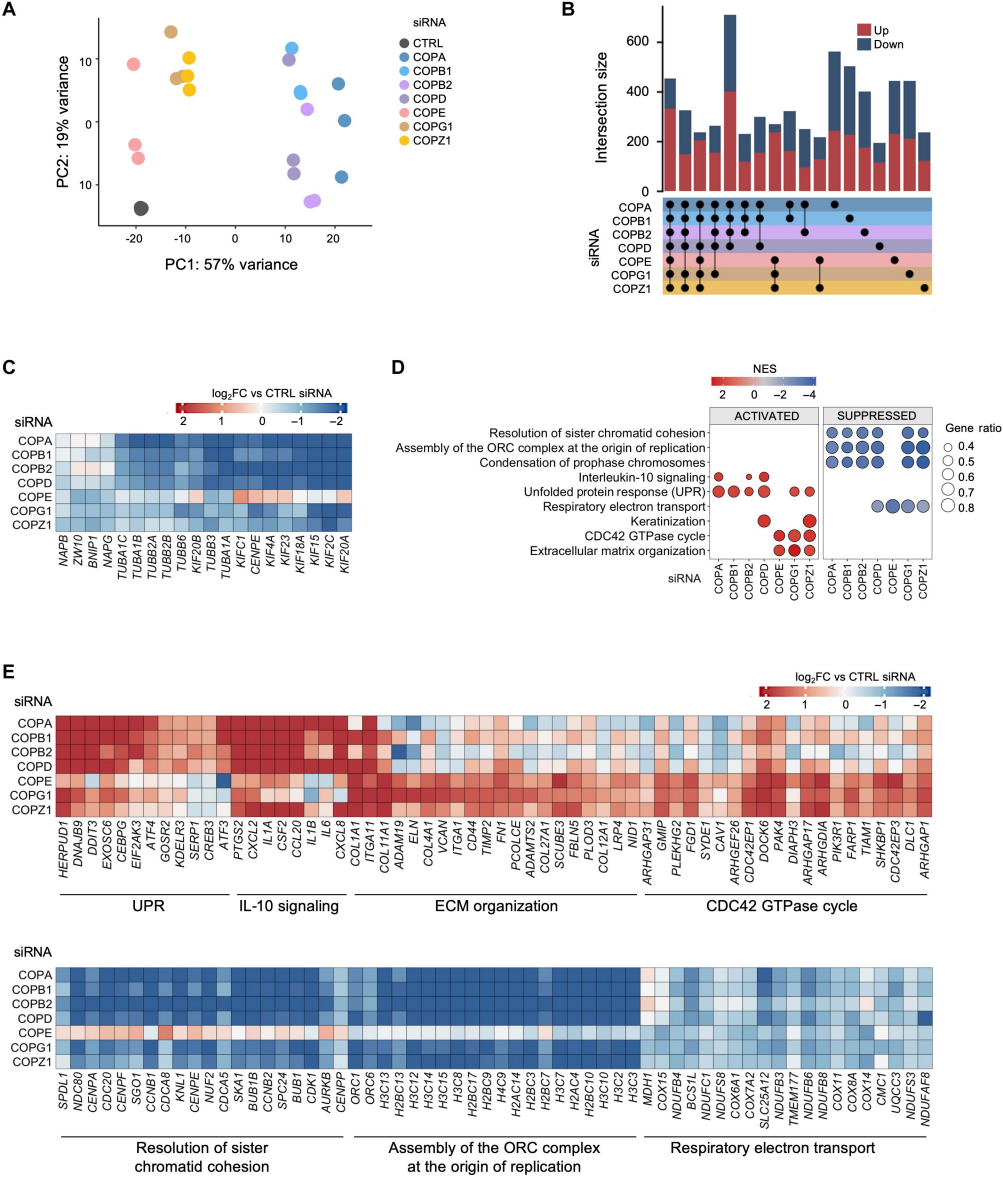
Transcriptomic changes after silencing of each COPI vesicle coat subunit by siRNA transfection. (**A**) PCA plot representing transcriptomic similarities and differences across samples, each in biological triplicates (GSE302417). (**B**) Combined analysis of RNAs affected by silencing of each individual COPI component. Transcripts whose levels either increased (Up) or decreased (Down) at least twofold compared to CTRL siRNA with *P*_adj_ < 0.01 were used for analysis. The upset plot shows joint changes for groups of 150 or more RNAs. (**C**) Effect of silencing each COPI subunit on mRNAs encoding proteins in the “COPI-dependent Golgi-to-ER retrograde traffic” pathway. (**D**) Top pathways identified by GSEA using the Reactome database performed on significantly changed RNAs (*P*_adj_ <0.01) after silencing each COPI component compared to CTRL siRNA. (**E**) Expression levels of selected mRNAs representing pathways identified in (D). In (C) and (E), the differences in RNA levels are represented as log_2_ fold change relative to the cells transfected with CTRL siRNA.

To gain insight into the cellular roles of each COPI protein member, we performed RNA-seq analysis after silencing individual COPI components with siRNAs directed at each of the encoding mRNAs (table S3; GSE302417). Principal components analysis (PCA) of the RNA-seq data identified those cells with silenced COPA, COPB1, COPB2, or COPD as a distinct group exhibiting similar transcriptomic alterations (Fig. 3A). This similarity was supported by the substantial overlap in the transcripts jointly elevated (red) or jointly reduced (blue) after silencing COPA, COPB1, COPB2, or COPD (Fig. 3B). At the same time, cells in which COPG1 or COPZ1 were silenced distributed together in PCA plots, while cells in which COPE was silenced had transcriptomes closest to those in the CTRL siRNA group (Fig. 3A).

COPI coat protein vesicles are primarily known for their function in retrograde transport between Golgi cisternae and from the Golgi to the ER (*10*). Thus, we examined whether a reduction in COPI subunits may influence this pathway. Focusing on the Reactome gene set “COPI-dependent Golgi-to-ER retrograde traffic,” we found that individually silencing each of the COPI proteins influenced the abundance of mRNAs encoding various kinesins and tubulins (Fig. 3C). This finding suggests that decreases in COPI proteins influence the levels of mRNAs encoding proteins important for cellular trafficking, and may impact broader aspects of cellular function. We investigated this possibility more systematically by GSEA using the Reactome database on the differentially expressed RNAs after individually silencing each component of COPI. As shown, silencing COPA, COPB1, COPB2, or COPD significantly increased the abundance of mRNAs encoding proteins involved in the unfolded protein response (UPR) and the production of inflammatory factors (Fig. 3, D and E), while silencing COPG1, COPZ1, or COPE instead increased the levels of mRNAs encoding proteins of the ECM and the CDC42 guanosine triphosphatase (GTPase) cascade of cell migration and polarity. In addition, silencing each COPI component, with the exception of COPE, decreased the production of mRNAs encoding regulators of DNA replication and cell cycle progression, and silencing any COPI component decreased the levels of mRNAs encoding proteins in the respiratory electron transport chain. These signaling processes—the UPR, the inflammatory response, ECM deposition, cell proliferation, and the mitochondrial electron transport chain—are all central to the senescent phenotype, suggesting that the individual COPI coat subunits can affect key senescence traits.

### Differential impact of individually silencing COPI subunits on NS and ETIS cell survival

**Fig. 4.**
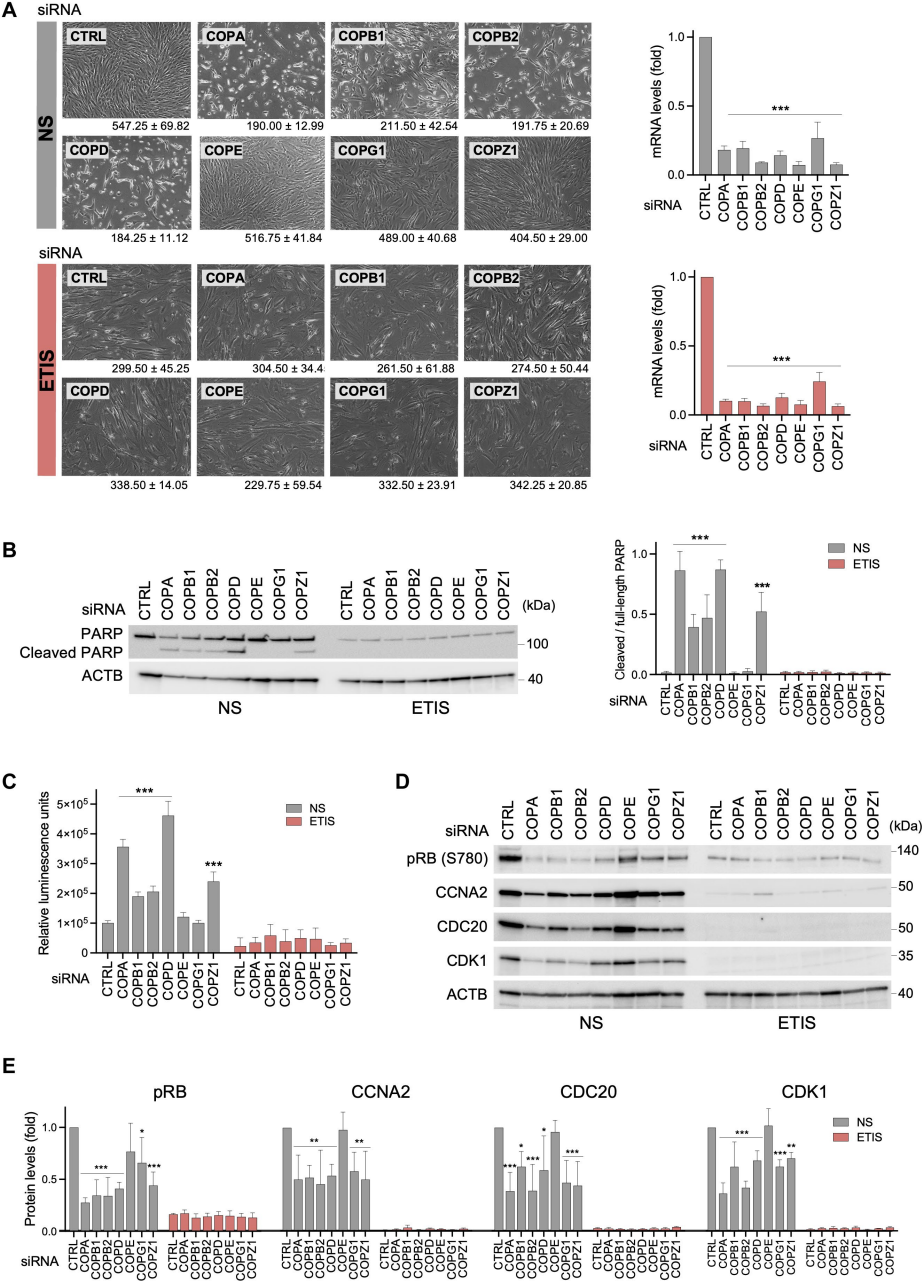
Distinct impact of silencing each subunit of the COPI complex upon NS and ETIS cells. WI-38 cells were either untreated (NS) and given DMSO or treated with 50 μM etoposide to trigger ETIS; on day 10 of culture with etoposide or DMSO, cells were transfected with either nontargeting siRNA (CTRL) or siRNAs directed at silencing the indicated subunits of the COPI complex. Assays were performed 72 hours after transfection. (**A**) Representative phase-contrast micrographs (left) depicting the appearance of each transfected population of NS fibroblasts (top) and ETIS fibroblasts (bottom). Scale bar, 200 μm. Quantification of the number of cells per field is indicated below each representative micrograph (*n* = 4). Transfection efficiency in each group was measured by RT-qPCR analysis (right); relative mRNA levels were normalized to *GAPDH* mRNA levels in each transfection group, and all were compared to the levels in CTRL cells. (**B**) Apoptosis was assessed by monitoring PARP cleavage, representative Western blots (left) and calculated ratio of cleaved to full-length PARP (right) (n = 3) are shown, and ACTB was included as a loading control. (**C**) Apoptosis was assessed by measuring caspase 3/7 activity (*n* = 5). (**D**) Representative Western blot analysis of cell cycle regulatory proteins encoded by mRNAs identified by RNA-seq analysis. (**E**) Quantification of the cell cycle regulatory protein levels represented in (D) from independent biological replicates (*n* = 3). The data were normalized to ACTB levels and compared to the levels in NS CTRL siRNA cells. Statistical significance was determined on the basis of the indicated number of independent experiments by an ANOVA followed by Dunnett’s post hoc test (*P < 0.05; **P < 0.01; ***P < 0.001) in comparison to CTRL siRNA in each group.

To investigate the influence of specific COPI coat components on cell senescence, we transfected WI-38 fibroblasts that were either proliferating (NS) or senescent (ETIS) with siRNAs targeting each individual COPI subunit. We found that reducing the levels of COPA, COPB1, COPB2, or COPD changed the appearance and decreased the number of NS WI-38 cells but did not have that effect on ETIS cells (Fig. 4A, left). RT-qPCR analysis verified the efficient silencing of each mRNA encoding a COPI component, excluding the possibility that these differences were due to variations in siRNA delivery (Fig. 4A, right). These reductions in cell number were also observed in other senescence models (RS and IRIS) of WI-38 populations in which each subunit of the COPI complex was silenced (fig. S3A), as well as in two other primary cell cultures, human umbilical vein endothelial cells (HUVECs) and hVSMCs, experiencing ETIS (fig. S4A). We thus set out to study whether the morphological changes observed were due to apoptotic death or reduced cell proliferation.

We first measured the cleavage of poly(ADP-ribose) polymerase 1 (PARP) (Fig. 4B) and the activity of caspases 3 and 7 (Fig. 4C), key markers of apoptosis, in these cells. In NS cells, silencing all COPI subunits, except COPE and COPG1, increased the abundance of cleaved PARP fragments and elevated the activity of caspase 3/7; these apoptotic indicators were especially prominent in cells transfected with COPA and COPD siRNAs (Fig. 4, B and C). In contrast, all ETIS cells exhibited no PARP cleavage and low caspase 3/7 activity, regardless of the transfected siRNA (Fig. 4, B and C). Our findings indicate that in NS cells, lowering the levels of COPA, COPB1, COPB2, COPD, and COPZ1 triggers apoptosis, while ETIS cells resist apoptosis despite reductions in COPI proteins.

We then focused on RNA-seq data showing that silencing each COPI protein (except COPE) decreased the levels of mRNAs encoding proteins involved in DNA replication and cell division (Fig. 3, D and E). The changes in levels of mRNAs encoding key proteins in these pathways, including *CCNA2*, *CCNB2*, *CDC20*, *CDK1*, *NUF2*, and *TOP2A* mRNAs, were validated by RT-qPCR analysis (fig. S5A). The effect of silencing COPI proteins on the cell cycle was further assessed by Western blot analysis of pRB phosphorylation at serine (S780) (a modification that is associated with cell proliferation and transition from the G_1_ phase to the S phase) and the abundance of CCNA2, CDC20, and CDK1 proteins (Fig. 4, D and E). These results revealed that silencing each COPI subunit (except COPE) led to significant reductions in the levels of mRNAs encoding proteins implicated in cell cycle progression in NS cells (Fig. 4, D and E). As anticipated, however, the levels of these cell cycle–related mRNAs and proteins decreased in ETIS CTRL compared to NS CTRL, and transfection of ETIS cells with COPI siRNAs did not further reduce their abundance.

In summary, silencing individual COPI subunits, except COPE, both increased apoptosis and reduced mRNAs encoding regulators of DNA replication and cell cycle progression in NS cells. We further found that these silencing interventions did not influence these traits in ETIS cells.

### Differential effect of silencing individual COPI proteins upon the UPR, autophagy, inflammatory signaling, and ECM

**Fig. 5.**
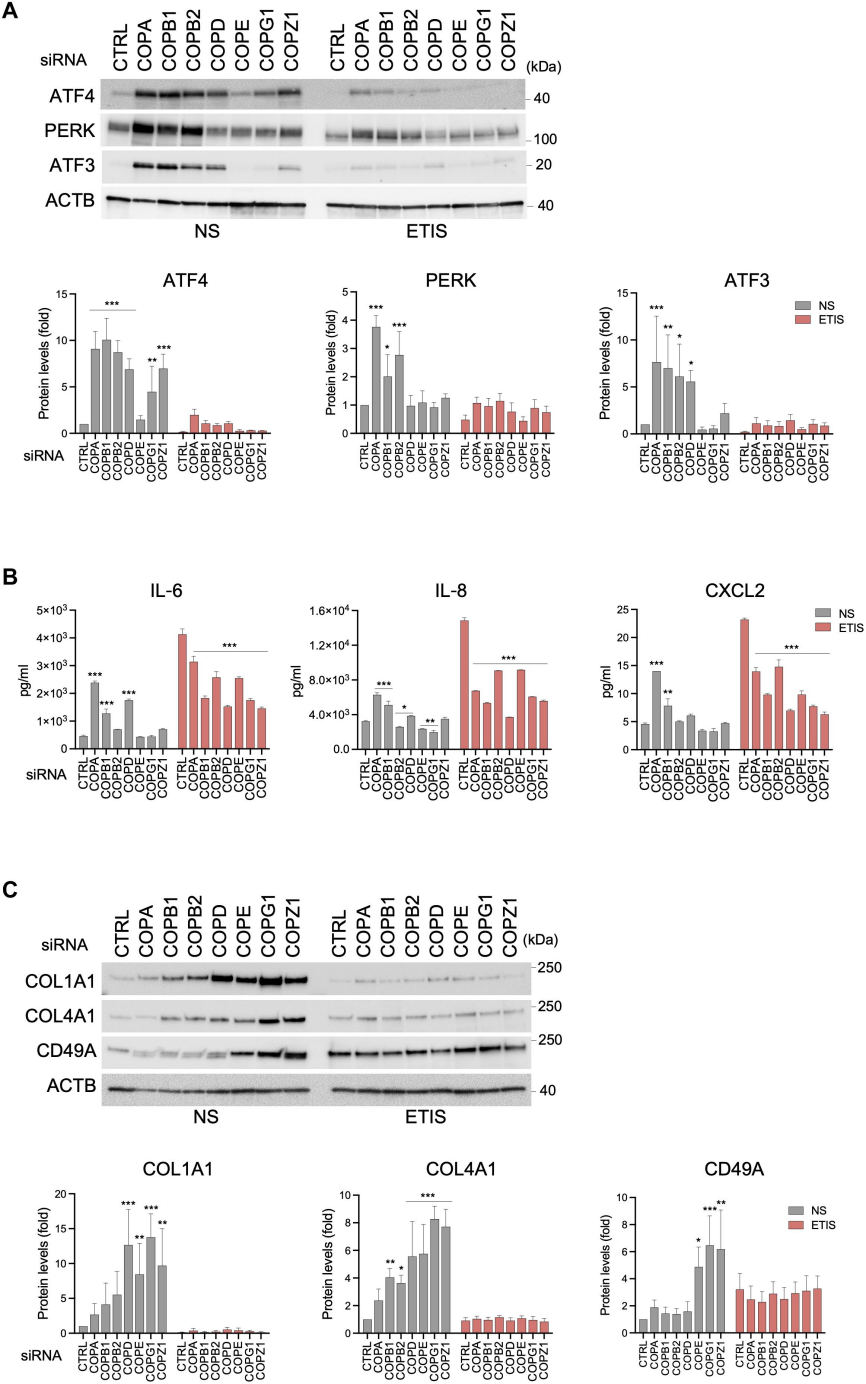
Impact on the UPR pathway and production of cytokines and ECM proteins in NS and ETIS cells after individually silencing each COPI component. WI-38 fibroblasts were processed as described in Fig. 4, whereupon additional analysis was performed. (**A**) The levels of ATF4, PERK, and ATF3 proteins were assessed by Western blot analysis (*n* = 3); representative images (top) and quantification (bottom) are shown. (**B**) Cytokine secretion was assessed by Bio-Plex analysis. Relative protein levels were normalized to the total protein concentration in each sample (*n* = 2). (**C**) The abundance of ECM proteins was assessed by Western blot analysis (*n* = 3); representative images (top) and quantification (bottom) are shown. In (A) and (C), the relative protein levels were normalized to ACTB levels and compared to the levels in NS CTRL siRNA cells. For all graphs, statistical significance was determined on the basis of the indicated number of independent experiments using an ANOVA followed by Dunnett’s post hoc test (*P < 0.05; **P < 0.01; ***P < 0.001) in comparison to CTRL siRNA in each group.

The transcriptomic data further prompted us to examine the hypothesis that silencing COPI subunits activated the UPR, inflammatory signaling, and ECM production (Fig. 3, D and E). We thus studied the UPR by evaluating key proteins implicated in this pathway encoded by mRNAs that were identified by RNA-seq analysis. As shown, the levels of ATF4, PERK, and ATF3, as assessed by Western blot analysis, were significantly elevated in NS cells after silencing COPA, COPB1, COPB2, or COPD; by contrast, their levels only increased slightly or remained unchanged after silencing COPE, COPG1, or COPZ1 (Fig. 5A). Given the central role of the transcription factor ATF4 in the ER stress response (*23*) and in light of the cross-talk between ER stress, ATF4 signaling, autophagy, and inflammation (*24*–*27*) and their complex interplay in senescence (*1*, *28*), we further examined how silencing COPI alters these processes in both NS and ETIS fibroblasts (Fig. 5A). Notably, in other senescence paradigms, the levels of ATF4 protein were also very low and changed minimally or remained unchanged under the same transfection strategy to silence COPI components, as observed in WI-38 cells rendered senescent by RS or IRIS and in HUVECs and hVSMCs experiencing ETIS (figs. S3B and S4B). These findings align with a recent report indicating persistent ER stress signaling and reduced production of ATF4 in senescent cells (*28*). Similar to silencing COPI proteins, treatment with the ER stress inducer tunicamycin only caused a small increase in ATF4 in ETIS compared to NS cells, further confirming that senescent cells did not mount a proper response to ER stress (fig. S5B).

We then evaluated autophagy by assessing the relative levels of LC3-I and LC3-II. In NS cells, silencing COPA, COPB1, COPB2, or COPD (but not COPE, COPG1, or COPZ1) elevated LC3-II/LC3-I ratios, as determined by Western blot analysis, and led to the accumulation of LC3-II autophagosome puncta, as determined by fluorescence microscopy imaging (fig. S6, A and B). By contrast, while senescent (ETIS) cells had higher overall LC3-II/LC3-I ratios than NS cells in the CTRL siRNA group, individually silencing COPI proteins did not substantially change these ratios (fig. S6A). These results agree with the aberrant autophagy reported in senescence (*29*) and further indicate that autophagy is altered in NS cells after individually silencing some COPI components, while in senescent (ETIS) cells, silencing COPI components did not further alter autophagy. While previous studies have reported the effect of the COPI complex on autophagy, our results indicate that different components of the complex have a distinct impact.

To assess changes in inflammatory signaling in these populations, we collected culture media 72 hours after transfecting siRNAs directed at each COPI protein and measured the levels of secreted cytokines using Bio-Plex. In NS cells, we found increased levels of interleukin-6 (IL-6), IL-8, and CXCL2 after silencing COPA, COPB1, or COPD (but generally not COPB2, COPE, COPG1, or COPZ1; Fig. 5B). In ETIS cells, the levels of these cytokines were elevated overall; however, silencing COPI subunits in ETIS cells reduced the levels of cytokines relative to those measured in CTRL siRNA cells. In addition, given the link between the inflammatory phenotype seen in COPA deficiency and the malfunction of the stimulator of interferon genes (STING) signaling pathway (*14*, *16*), we assessed the extent of phosphorylation of TANK-binding kinase 1 (TBK1), a key downstream effector of STING activation that propagates inflammatory signaling. The results showed a significant increase in TBK1 phosphorylation at alanine 172 [pTBK1 (A172)] in NS WI-38 cells after silencing COPA, COPB1, COPB2, or COPD, while silencing COPG1 or COPZ1 only slightly increased pTBK1 levels, and silencing COPE did not increase the pTBK1 signal at all (fig. S7A). In ETIS cells, pTBK1 signals were very low compared to NS cells, and they remained unchanged after silencing COPI components. These results support a role for the STING pathway in inflammatory signaling triggered after silencing selected COPI components and indicate that the involvement of STING was markedly less prominent in ETIS.

Moreover, silencing several COPI subunits significantly increased the levels of mRNAs encoding ECM components in NS cells (Fig. 3, D and E). Here, Western blot and RT-qPCR analyses confirmed the increase in the levels of COL1A1, COL4A1, and ITGA1/CD49A in NS cells, as well as in the corresponding mRNAs, particularly after silencing COPE, COPG1, or COPZ1 (Fig. 5C and fig. S7B). In ETIS, silencing individual COPI proteins did not markedly change the levels of these ECM components when compared to CTRL siRNA. Together, these findings indicate that in NS cells, silencing COPA, COPB1, COPB2, and COPD increased ATF4 production and modulated autophagy and inflammatory signaling; on the other hand, silencing COPE, COPG1, and COPZ1 had a minimal impact on ATF4 levels, autophagy, and signaling, but instead, it increased the levels of ECM constituents. Our collective results further indicate that these responses are impaired in senescence.

### Impact of silencing COPI proteins on mitochondria function

**Fig. 6.**
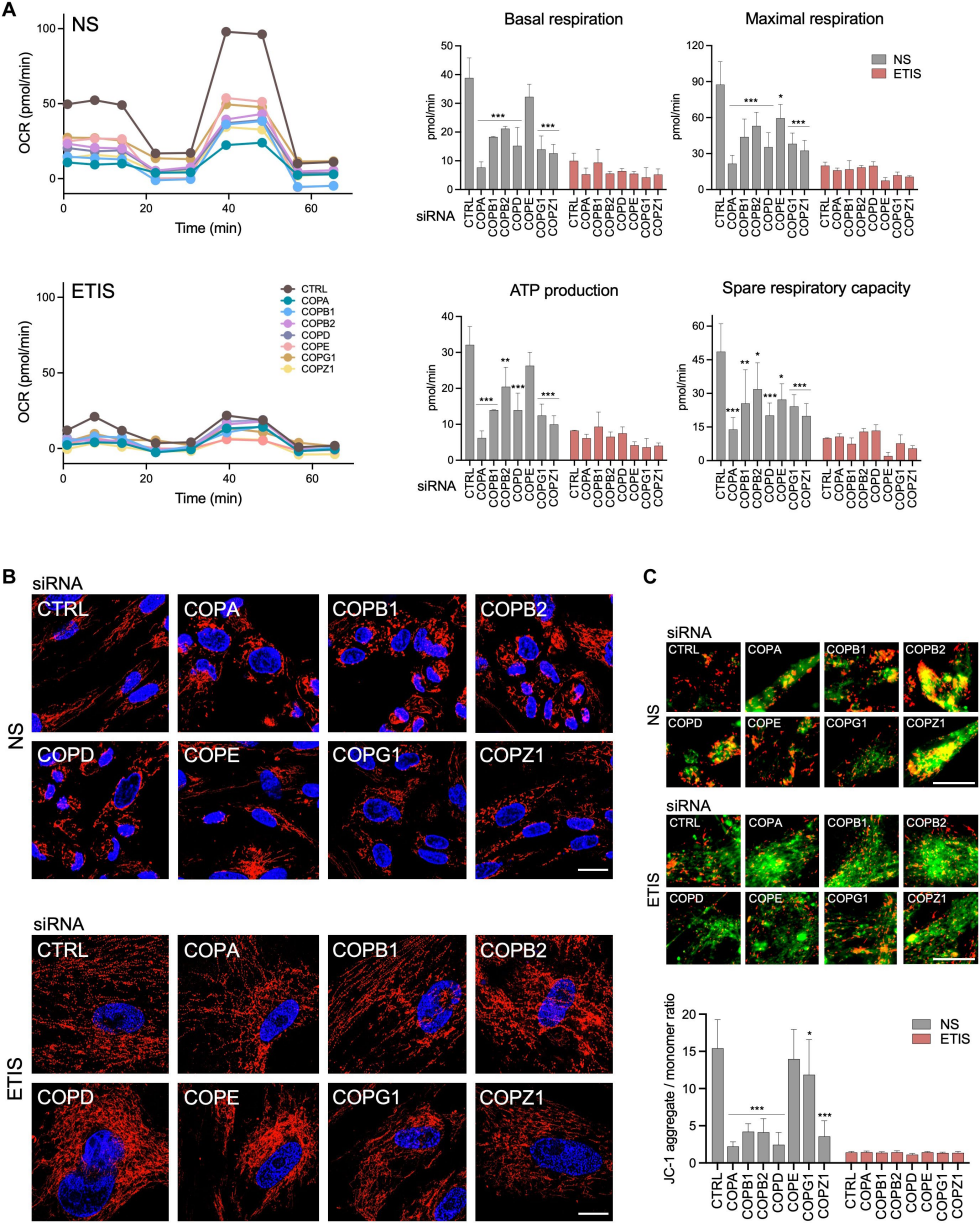
Effect of silencing the COPI coat subunits on mitochondrial function in NS and ETIS cells. WI-38 cells were processed as described in Fig. 4. (**A**) Mitochondrial respiratory activity was assessed by measuring cellular OCR in live cells using a Seahorse XF Cell Mito Stress test assay. These parameters were evaluated: basal respiration, maximal respiration, ATP production, and spare respiratory capacity. The values were normalized to the protein concentration in each sample (*n* = 3). (**B**) Representative confocal immunofluorescent micrographs depicting changes in mitochondrial morphology as visualized by analysis using an antibody that recognizes the mitochondrial membrane with TOM20 (red); nuclei (blue) were labeled with DAPI. Scale bars, 20 μm. (**C**) Mitochondrial membrane potential was evaluated by using the JC-1 dye. Representative fluorescent micrographs showing intensities of red fluorescence (JC-1 aggregates) and green fluorescence (JC-1 monomers) in each experimental condition (top). Fluorescent red and green signals were quantified, and relative intensities were represented as JC-1 aggregate/monomer ratios to assess mitochondrial health (*n* = 6) (bottom). Scale bars, 50 μm. In (A) and (C), statistical significance was determined by an ANOVA followed by Dunnett’s post hoc test (*P < 0.05; **P < 0.01; ***P < 0.001) in comparison to CTRL siRNA in each group.

We next investigated the consequences of silencing COPI components on the mitochondrial electron transport system, another key pathway uncovered by RNA-seq analysis (Fig. 3, D and E). Mitochondrial function was evaluated by measuring the oxygen consumption rate (OCR) in cells using an Agilent Seahorse XFe24 Analyzer instrument 72 hours after transfection (Fig. 6A). As observed, in NS cells, silencing each COPI protein markedly decreased the OCR compared to CTRL siRNA; this reduction was evident when evaluating basal respiration, ATP (adenosine 5′-triphosphate) production, maximal respiration, and spare respiratory capacity. In contrast, ETIS cells showed overall lower OCR compared to NS cells, and silencing COPI components did not further reduce these parameters (Fig. 6A).

To further evaluate mitochondria in this paradigm, we carried out confocal microscopy after immunostaining with an antibody that recognizes the mitochondrial membrane receptor protein TOM20. This analysis revealed a fragmented, aggregated, and enlarged mitochondrial compartment consistent with mitochondrial dysfunction after individually silencing COPI subunits (particularly COPA, COPB1, COPB2, or COPD) in NS cells (Fig. 6B). In contrast, all ETIS cells displayed an enlarged and dispersed mitochondrial compartment relative to NS CTRL siRNA cells, in agreement with the accumulation of dysfunctional mitochondria in these cells, as previously reported in senescence models and aged cells (*2*, *30*–*33*), and in agreement with the mitochondrial dysfunction observed after silencing COPI proteins in yeast and human neuroblastoma cells (*34*, *35*).

We used the dual-emission fluorescent dye JC-1 to assess the mitochondrial membrane potential. JC-1 forms red aggregates in healthy mitochondria with intact membrane potential, while it emits green fluorescence in monomeric form when the mitochondrial membrane potential is disrupted. High red-to-green fluorescence signal ratios in the NS CTRL siRNA sample indicated healthy mitochondria in these cells (Fig. 6C). On the other hand, significantly lowered red/green ratios were seen after silencing COPA, COPB1, COPB2, COPD, or COPZ1 in NS cells, indicating decreased membrane potential in these cell populations; silencing COPE or COPG1 did not reduce these ratios markedly relative to CTRL siRNA cells. All ETIS cells displayed lower red/green ratios compared to NS CTRL siRNA cells, with no effect after silencing COPI components, further supporting the presence of dysfunctional mitochondria in senescent cells. These results collectively indicate that individually silencing COPI proteins disrupts mitochondrial function in NS cells, while in ETIS cells, mitochondrial activity is impaired, and silencing COPI proteins has no further impact.

### COPI proteins associate with proteins in the endosomal system

Given the involvement of COPI in the formation, cargo selection, and intracellular trafficking of transport vesicles, together with our observations of diverse effects of COPI subunits on cellular functions, we explored possible interactions between COPI subunits and other proteins involved in intracellular transport across intracellular compartments. We carried out IP of WI-38 whole-cell lysates using antibodies targeting individual COPI proteins, followed by MS-based proteomic analysis [table S4; MassIVE MSV000098471 (PXD065972); https://massive.ucsd.edu/ProteoSAFe/dataset.jsp?task=a4ea9ff098664993ae132cddea18bd5b]. We focused on IP reactions using antibodies that recognized COPB1, COPE, and COPZ1, as these three proteins represent members of COPI protein subsets with various effects on cell transcriptomes and function (Fig. 3); the antibodies enabled efficient and clean IP of COPB1, COPE, and COPZ1 (fig. S8A). Among the proteins enriched in the respective IPs relative to control immunoglobulin G (IgG) IP [log_2_FC (log_2_ fold change) > 5; *P*_adj_ <0.05], we selected those that were members of the “Vesicle-Mediated Transport” GO Biological Process (BP) category in the Molecular Signature Database (*36*). We found 115 proteins related to vesicle transport associated with the COPI complex (fig. S8B).

**Fig. 7.**
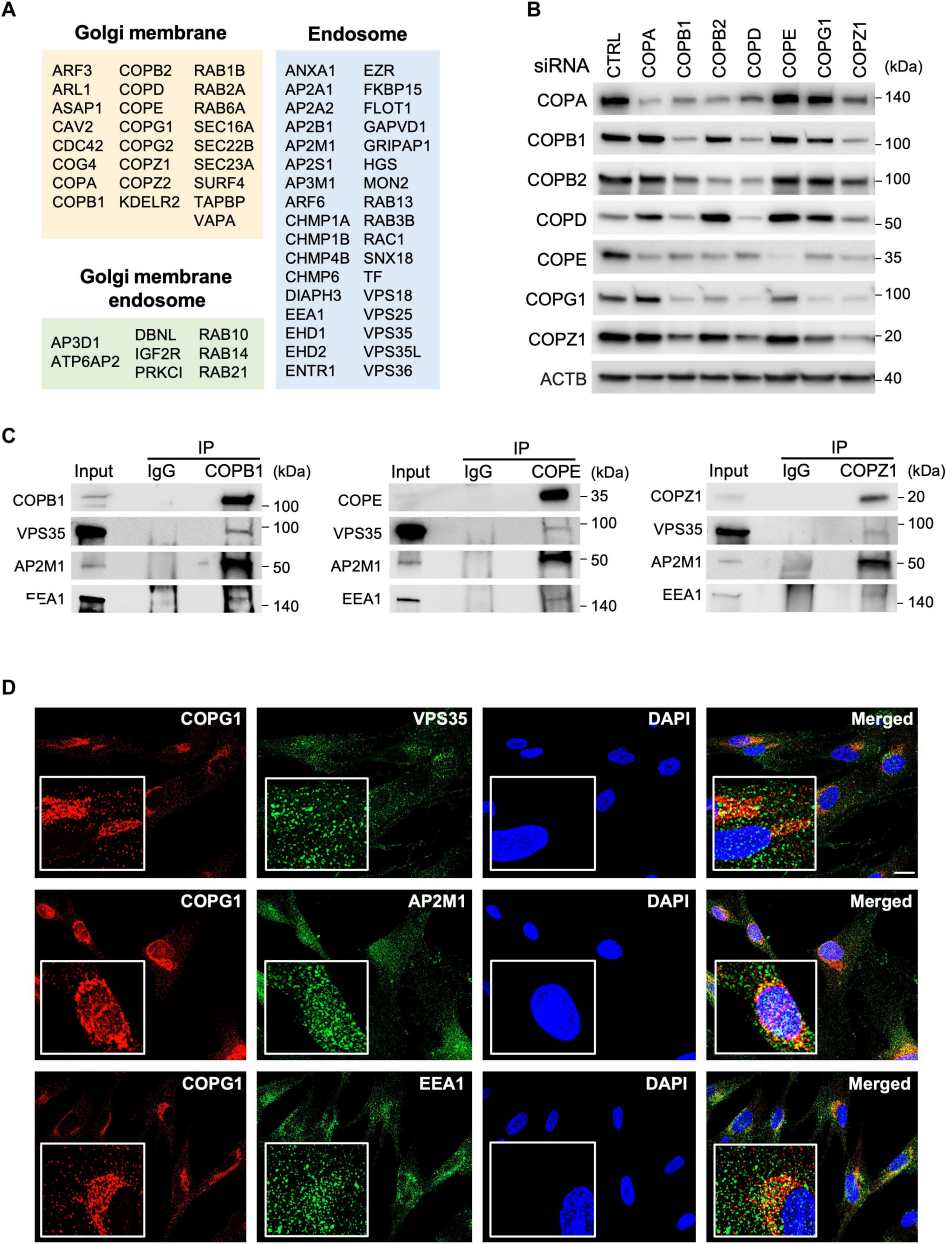
COPI complex–interacting proteins. (**A**) Lists include proteins that were enriched by MS analysis after IP of COPI components and were found to be in GO CC “Golgi Membrane” and/or “Endosome” datasets. (**B**) Effect of silencing each COPI component on the expression levels of the other components of the COPI complex. (**C**) Western blot analysis of proteins VPS35, AP2M1, and EEA1, selected among the endosome proteins listed in (A), after IP of COPB1, COPE, or COPZ1. (**D**) Representative confocal immunofluorescent images after costaining COPG1 with endosomal system proteins VPS35, EEA1, and AP2M1. Scale bar, 20 μm.

COPI-coated vesicles are best known for facilitating Golgi-to-ER retrograde transport, but they also play a lesser-understood role in endocytic trafficking (*12*, *37*). Given the initial observation that prompted this study, namely the reduction in EV uptake by recipient cells after reducing the abundance of COPI proteins (Fig. 1, D and E), and given that endocytosis is a principal mechanism for cellular uptake (*38*), we used the GO Cellular Components (CC) “Golgi Membrane” and “Endosome” gene sets to identify COPI complex–associated proteins of the Golgi membrane and the endosomal system. Multiple proteins related to endosomal trafficking coimmunoprecipitated with the COPI complex (Fig. 7A). Notably, all COPI subunits were found among the COPI-interacting proteins of “Golgi Membrane,” confirming the interaction of these subunits in the COPI complex and demonstrating the effectiveness of the IP strategy. Western blot analysis of COPI subunits after individually silencing each COPI protein showed that decreasing COPB1, COPD, and COPZ1 generally led to reductions in the levels of other subunits (Fig. 7B and fig. S8C). On the other hand, silencing COPE did not affect the expression of other components of the complex, but COPE levels declined by silencing any COPI subunit; silencing other COPI proteins had varying effects on the expression levels of all subunits. The variable impact of silencing some COPI subunits upon the abundance of other COPI proteins agrees with an earlier report (*14*) and further supports the notion that COPI proteins may have a different influence on the structure and function of the COPI complex.

Among the proteins of the endosomal system that were identified by IP-MS to associate with the COPI complex, several proteins were implicated in the vesicle trafficking network between intracellular organelles, including components of AP-2 (adaptor protein complex 2), members of vacuolar protein sorting (VPS), and proteins of the Rab GTPase family (Fig. 7A). To confirm these results, we selected three representative proteins: adaptor-related protein complex 2 subunit μ1 (AP2M1), involved in clathrin-mediated endocytosis but also reported in the membranes of lysosomes, ER, and Golgi (*39*); the retromer complex component VPS35, known for regulating the retrograde transport vesicles from endosomes to the Golgi network (*40*); and early endosome antigen 1 (EEA1), which facilitates fusion and maturation of early endosomes (*41*). Western blot analysis showed the enrichment of EEA1, AP2M1, and VPS35 proteins after IP of all COPI subunits tested (COPB1, COPE, and COPZ1) but not in the control IgG IP samples (Fig. 7C), supporting the specificity of these interactions. These associations were further confirmed and localized by confocal immunofluorescence microscopy. COPG1 signals largely colocalized with COPE and COPB1 signals, with large overlaps in the fluorescence, confirming the association of subunits in the COPI complex and their predominant colocalization near the nucleus (fig. S9). Immunofluorescence analysis using antibodies that recognized COPG1 and the endosomal proteins EEA1, AP2M1, and VPS35 revealed puncta of overlapping and nonoverlapping signals, indicating that the endosomal proteins tested are in partial proximity with the COPI complex and suggesting a dynamic and perhaps space-restricted interaction among them (Fig. 7D). On the basis of our findings, we propose that proteins of the COPI complex associate with transporters of the endosomal network, connecting vesicular trafficking between the Golgi and endosomes. We further propose that the reduction in COPI components with senescence affects EV uptake from the intracellular space and impairs intracellular communication across organelles.

## DISCUSSION

Senescent cells are characterized by changes in the morphology and function of organelles. While the dysfunction of mitochondria, lysosomes, and the ER in senescence has been studied extensively (*42*), the distorted Golgi organization and the Golgi-associated intracellular trafficking in senescence remain poorly understood. Intracellular trafficking involves a complex network of membrane-enclosed transport vesicles that move molecules between subcellular regions and play key roles in maintaining cellular homeostasis and connecting the cell with its environment (*43*). Vesicular transport mediates active communication among various compartments such as the plasma membrane, endosomes, lysosomes, Golgi apparatus, ER, and nucleus. This intracellular communication regulates cell functions including protein synthesis and cell signaling, as well as interactions with the extracellular space through secretion and uptake of molecules. Evidence is accumulating that cellular trafficking and organelle communication are disrupted in senescent cells and in pathologies in which senescent cells accumulate (*44*, *45*).

Our study was prompted by the discovery through an RNAi screen that silencing proteins in the COPI complex diminished the uptake of EVs by recipient cells (Fig. 1, B to E). These findings were somewhat unexpected, given that COPI proteins coat vesicles trafficking from the Golgi membrane destined for other subcellular organelles, but they aligned with previous findings of reduced transferrin uptake and disruption of endosomal protein sorting and transport after depleting various COPI subunits (*12*, *46*–*48*). Our data also agreed with earlier reports that COPI proteins were essential for virus infection and bacterial invasion into host cells (*17*, *18*, *49*, *50*). The exact mechanisms by which proteins in the COPI complex contribute to cellular uptake are not fully understood and are likely multifaceted. Earlier work demonstrated that depleting components of the COPI complex [e.g., by siRNA-mediated COPA silencing in HeLa cells and by degradation of a temperature-sensitive COPE subunit in ldlF Chinese hamster ovary cells (*18*, *47*)] decreases the abundance of heparan sulfate on the cell surface, indicating a role of COPI in heparan sulfate–mediated cellular internalization. Furthermore, the dispersal and expansion of the Golgi structure observed in cells with silenced COPI proteins (Fig. 1F) could lead to mislocalization and reduced levels of other essential molecules needed for effective cellular uptake. In keeping with the enlarged Golgi compartment seen after silencing COPI proteins (Fig. 1F), senescent cells, which express modest but consistently lower levels of several COPI subunits (Fig. 2, G to I, and fig. S2A), also exhibited a larger Golgi apparatus (Fig. 2, C and D, and fig, S1, A and B) and reduced cellular uptake (Fig. 2, E and F) (*4*, *5*, *20*, *51*, *52*). Further research is needed to fully elucidate the specific roles of COPI complex proteins in cellular uptake processes.

To understand the cellular functions of each COPI member, we performed RNA-seq analysis after silencing individual COPI proteins in NS WI-38 fibroblasts. These interventions showed distinct clusters of effects on PCA plots: Silencing COPA, COPB1, COPB2, or COPD yielded transcriptomes that clustered together (Fig. 3A), and silencing COPG1 or COPZ1 produced separate transcriptomes clustering in close proximity, while silencing COPE generated yet another set of transcriptomes closest to those seen in the CTRL siRNA population (Fig. 3B), suggesting that the encoded proteins contributed in three distinct ways to cellular processes.

In NS cells, reducing COPA, COPB1, COPB2, or COPD increased the abundance of mRNAs encoding proteins involved in the UPR, including the transcription factor ATF4, a main effector of the integrated stress response triggered by ER stress (*53*). Consequently, these cells displayed phenotypes triggered by ER stress, including altered autophagy, cytokine secretion, mitochondrial dysfunction, cell cycle arrest, and apoptosis (Figs. 3 to 6 and fig. S6). While these modified processes have been linked to ER stress, they have also been reported in cells deficient in individual COPI subunits (*14*, *15*, *34*, *35*, *54*–*56*). Notably, the mutation in COPA was found to impair ER-Golgi trafficking, leading to ER stress and increased cytokine production in COPA syndrome (*15*). Other studies have shown that defective retrograde transport triggered by COPA mutation disrupts the Golgi-to-ER retrieval of the immune signaling protein STING, leading to its accumulation in Golgi compartments. This results in overactivation of the STING pathway, increased IFN-I (type I interferon) signaling, and inflammation in COPA syndrome (*14*, *16*). Similar IFN-I activation was observed after genetic deletion of COPD or COPG1 but not after deletion of COPE (*14*). Our findings of UPR activation and a significant increase in TBK1 phosphorylation (a key downstream mediator of STING activation) following the silencing of COPA, COPB1, COPB2, or COPD indicate a prominent role of these COPI subunits in the STING pathway. The increase in pTBK1 was less pronounced when COPG1 or COPZ1 was silenced, and no effect was observed in cells silenced with COPE (fig. S7A). However, the exact manner in which each COPI subunit is implicated in the ER stress response is not fully understood. A plausible link is that decreases in the abundance of any of these four subunits cause an imbalance in the sorting of cargo in vesicles, leading to the accumulation of misfolded proteins destined for the ER. While these processes are already impaired in senescence, lowering the abundance of these COPI subunits in senescent fibroblasts did not further exacerbate these aberrant processes (Figs. 4 to 6). Senescent cells displayed suppressed ATF4 production, persistent ER stress signaling, and a reduced integrated stress response (*28*), as well as resistance to apoptosis resulting from reduced caspase 3/7 activity levels (*57*). Although these features of senescent cells likely contribute to the refractory response of senescent cells to silencing COPI proteins, the exact mechanisms involved need further study. Additional work is also needed to reconcile our results that COPI silencing did not significantly increase apoptosis in senescent cells, in light of the findings of McHugh *et al.* (*58*) indicating that reducing COPI function induced apoptosis in IMR-90 fibroblasts. Perhaps the extent of COPI reduction, the degree of damage leading to senescence, the timing of analysis, or other variables in culture conditions explain the different responses in the two paradigms.

Silencing COPG1, COPZ1, or COPE in NS fibroblasts had a lesser impact on the cellular stress response pathway than the silencing of COPA, COPB1, COPB2, and COPD. Instead, reductions in COPG1, COPZ1, or COPE increased the levels of mRNAs encoding proteins in the ECM and the CDC42 GTPase cycle, indicating a specialized role of these subunits in the function of the COPI complex. While previous reports have suggested that COPI proteins are involved in regulating the production and transport of ECM components like collagen (*59*), further experimentation is needed to fully understand the mechanism and meaning of this regulation. Silencing COPE resulted in the smallest transcriptomic changes compared to other subunits, with minimal or no impact on processes related to the stress response or the cell cycle. Although COPE is not essential for cell viability (*60*), lowering COPE levels led to decreased uptake of EVs and increased levels of ECM components. In addition, reducing COPE levels did not affect the abundance of other subunits, while silencing any of the other COPI subunits decreased the expression of COPE (Fig. 7B). This evidence suggests that COPE has a specific function in cellular uptake but plays a generally supportive role in the COPI complex, which agrees with its being somewhat “dispensable” in our experimental system, in agreement with earlier observations (*14*). Given that endocytosis is a primary pathway for EV uptake into endosomal compartments (*61*), these data support the involvement of COPE specifically in the regulation of endocytic trafficking. Earlier studies showed that degradation of COPE compromised endosomal trafficking (*47*).

The involvement of COPI coat proteins in processes other than retrograde Golgi-to-ER trafficking, including the endosomal system, was reported earlier (*49*, *62*). It has been suggested that the COPI complex may play a role in vesicular trafficking for communication between multiple compartments (*48*). For example, COPI subunits were shown to interact and colocalize with the protein VPS27 for a role in endosomal protein sorting to the multivesicular body compartment in yeast (*12*). We found an association of multiple proteins of the endosomal system with the COPI complex (Fig. 7A), further supporting the idea that subunits of the COPI complex may facilitate vesicular trafficking between different cellular compartments. The observed reduction in EV uptake by senescent cells in our study possibly reflects the diminished endocytosis in senescent cells previously reported (*52*, *63*). In this regard, it would be particularly important to elucidate the precise stoichiometry of each COPI component in each COPI complex, as it may change dynamically over time and in each subcellular space. This information could help explain the different functions for COPI components identified here.

**Fig. 8.**
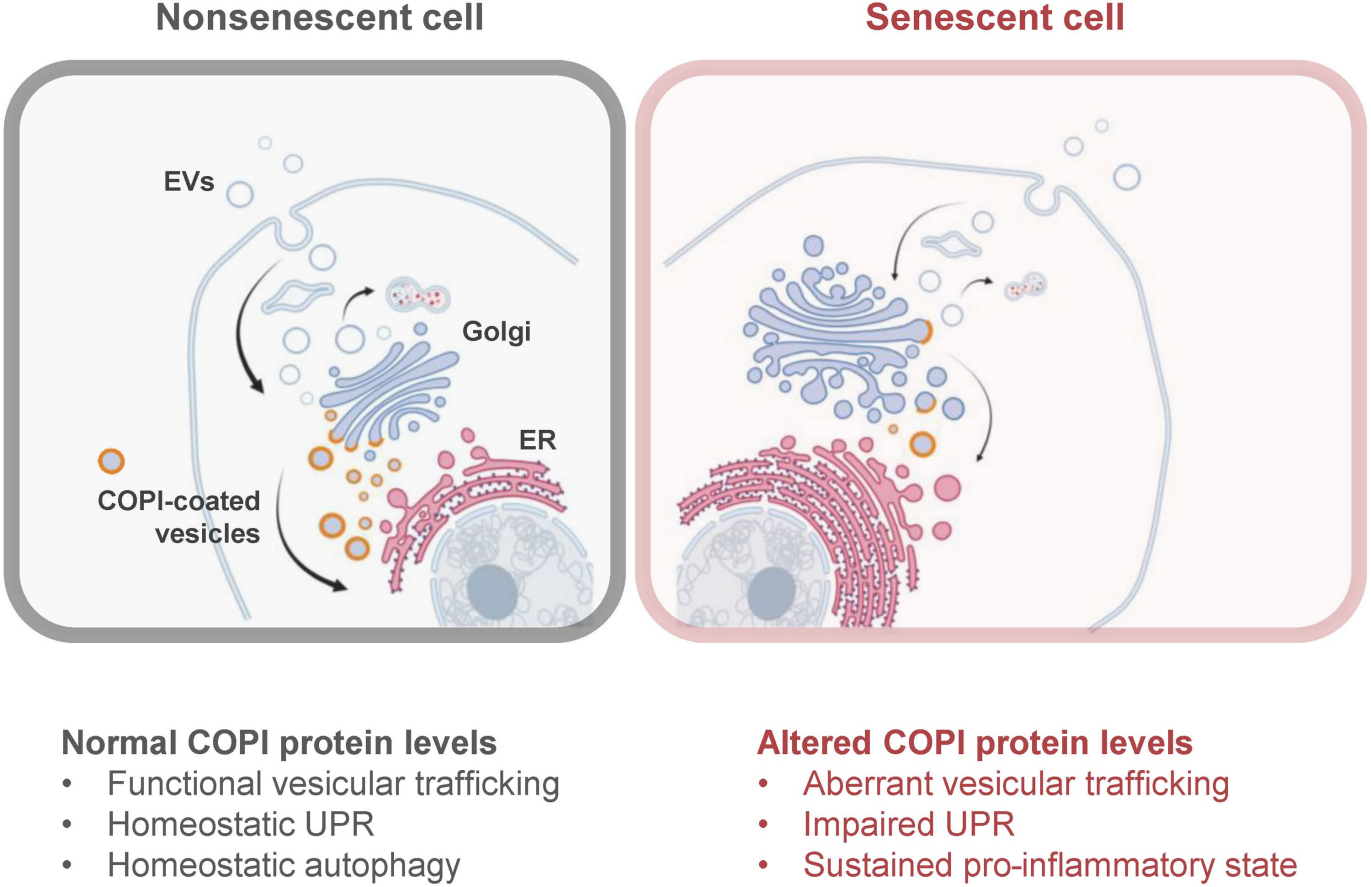
Model. Protein components of the COPI coatomer complex were found to contribute to the internalization of EVs. In NS cells, COPI components were important for maintaining the homeostasis of intracellular vesicle trafficking, autophagy, UPR, and apoptosis. In senescent cells, where COPI components are overall less abundant, intracellular vesicle trafficking was impaired, the Golgi was enlarged, and there were aberrant autophagy, UPR, and apoptosis. Senescent cells are largely unresponsive to reductions in COPI proteins, suggesting that COPI-mediated transport functions are already compromised during senescence.

In conclusion, our results underscore key roles for subsets of COPI coatomer components in intracellular transport, organelle function, and EV uptake—processes that are impaired in senescent cells. Silencing individual COPI subunits had a negligible impact on senescent cells but disrupted autophagy, mitochondrial respiration, inflammation, and cell cycle progression in NS cells, highlighting an attenuation of COPI function in senescence. We propose that dynamic COPI regulation critically maintains organelle function in NS cells, and its decline during senescence contributes to the disruption in homeostasis and intercellular communication seen in age-related decline and disease (Fig. 8).

### Study limitations

Our study has uncovered that individual COPI proteins contribute differently to maintaining cell homeostasis and that their function is compromised in cellular senescence, but we recognize several limitations. First, because of the pleiotropic activity of COPI proteins, it was not possible to define the precise mechanism of action of each COPI protein; we found that COPA, COPB1, COPB2, and COPD are involved in the UPR and inflammation and that COPE, COPG1, and COPZ1 control the ECM, but elucidating the distinct molecular function of each protein awaits future investigation. Second, while COPI has been involved in maintaining homeostatic autophagy (*46*), understanding the precise involvement of specific COPI components in regulating autophagy also warrants dedicated future study. Third, we identified interactions of COPI proteins with proteins involved in Golgi trafficking and the endosomal system, but these results also necessitate further in-depth investigation to link each COPI protein to specific intracellular transport networks. Future work toward these goals must leverage advanced microscopic analyses and specialized subcellular fractionation strategies to define the interactions of COPI constituents with ER, lysosomes, mitochondria, endosomes, and other subcellular components. This information will help delineate the impact of COPI components upon cytoplasmic trafficking to preserve homeostatic interorganellar communication.

## MATERIALS AND METHODS

### Cell culture, senescence induction, and transfection

Human diploid WI-38 lung fibroblasts (Coriell Institute for Medical Research: ID AG06814) were cultured in Dulbecco’s modified Eagle’s medium (Gibco) supplemented with 20% fetal bovine serum (FBS), 1% penicillin/streptomycin, and 1% nonessential amino acids. Normal, pooled, primary HUVECs [American Type Culture Collection (ATCC), PCS-100-013] were cultured in vascular cell basal medium (ATCC, PCS-100-030) supplemented with Endothelial Cell Growth Kit-VEGF (ATCC, PCS-100-041) in a humid incubator at 5% CO_2_. hVSMCs were obtained from LifeLine Cell Technology (no. FC-0031) and maintained in VascuLife SMC Medium Complete Kit (LifeLine Cell Technology, no. LL-0014) following the manufacturer’s protocol. Cells were kept in a humid incubator at 5% CO_2_ and 37°C. In WI-38 cells, senescence was induced by treating cells with 50 μM etoposide every 3 days for 10 days (ETIS), by exposure to 10 grays (Gy) of ionizing radiation and culture for an additional 10 days to achieve IRIS, or by culture until replicative exhaustion (RS) at population doubling level (PDL) 54. HUVECs were induced into senescence (ETIS) by treatment with 10 μM etoposide for 3 days, followed by medium renewal without etoposide until day 9. hVSMCs were rendered senescent (ETIS) by treatment with 50 μM etoposide for 10 days. NS cells were maintained in a proliferative state below PDL 25 for WI-38 cells and PDL 2 or 3 for HUVECs and hVSMCs. NS cells were treated with dimethyl sulfoxide (DMSO) when compared with ETIS cells. Senescence was validated by measuring the activity of SA-β-Gal (Cell Signaling Technology) and by quantifying the production of mRNAs encoding SASP factors and canonical senescence markers, such as p16/CDKN2A, p21/CDKN1A, and DPP4. Transfections were performed using Lipofectamine RNAiMax (Thermo Fisher Scientific) in Opti-MEM (Gibco) containing 20 to 30 nM individual siRNAs (Dharmacon) added to the cells; the medium was replaced 6 hours later with regular growth medium, and cells were harvested 72 hours after that.

### EV isolation, labeling, and uptake

EVs were isolated and labeled as described (*64*). Briefly, cells were cultured in Dulbecco’s modified Eagle’s medium containing 10% exosome-depleted FBS (Gibco); the medium was centrifuged at 500*g* for 10 min at 25°C, at 2000*g* for 20 min, and at 15,000*g* for 40 min at 4°C. After ultracentrifugation at 120,000*g* for 16 hours at 4°C, pelleted EVs were resuspended in 4 ml of filtered PBS, ultracentrifuged at 126,000*g* for 1 hour at 4°C, and washed with phosphate-buffered saline (PBS). To label EVs, 10^10^ EV particles were resuspended in PKH26 red fluorescent dye (Sigma-Aldrich), incubated for 5 min on ice, and quenched with 5 ml of exosome-depleted FBS and 2 ml of 10% bovine serum albumin (Gibco). PKH26-labeled EVs were further purified by ultracentrifugation at 190,000*g* through a 0.971 M sucrose solution for 2 hours at 4°C. The EV size, concentration, and labeling efficiency were assessed using NanoSight (Malvern) and NanoAnalyzer (NanoFCM Co., Ltd.) instruments. To monitor EV uptake, cells were incubated with an equal volume of fluorescently (PKH26) labeled EVs for 24 hours and then analyzed by flow cytometry (Canto II flow cytometer or BD SORP FACSAria Fusion, BD Biosciences, and FlowJo software, version 10.2) and fluorescence microscopy (Keyence BZ-X710).

### RNAi screening of EV uptake

An RNAi screen was carried out using a whole-genome library targeting ∼21,597 genes with three independent siRNAs per gene (Silencer Select Human Genome siRNA Library V4, Ambion). For reverse transfection, each individual siRNA (400 nM in 2 μl) was robotically preprinted per well into 384-well black microplates with clear bottoms (Greiner). Next, 20 μl of serum-free media with Lipofectamine RNAiMax (Thermo Fisher Scientific) was added to each well and incubated for 45 min at 25°C to form siRNA-lipid complexes. Then, 500 cells in 20 μl of media containing 20% FBS were added per well and cultured for 72 hours. To assess the effect of each siRNA-mediated silencing on uptake, the transfected cells were incubated with EVs (6 × 10^6^ particles per well) fluorescently labeled with PKH26 for an additional 24 hours. The cells were fixed, stained with 4′,6-diamidino-2-phenylindole (DAPI), and evaluated by fluorescence imaging (ImageXpress Automated Cell Imaging System, Molecular Devices). The fluorescence intensity in each cell (determined by DAPI counts) in each well was acquired using quantitative image analysis. The fluorescent signal of each well with siRNA-transfected cells was normalized to the signal of cells transfected with CTRL siRNA. To rank genes whose silencing altered the cellular uptake of EVs, the median seed-corrected *z*-score was calculated as described (*65*). Candidates with *z-*score cutoff >1 and <−1 were considered as potential contributors for altered uptake.

### Western blot analysis

WI-38 cells were harvested by scraping into PBS; after gently pelleting, cells were dissolved in lysis buffer (50 mM Hepes, 2% SDS, and protease/phosphatase inhibitors), incubated at 95°C for 5 min, and sonicated for 5 min. Protein concentrations were measured (Pierce BCA Protein Assay Kit, Thermo Fisher Scientific), and protein aliquots (2 to 30 μg per sample) were combined with 4× Laemmli buffer containing 10% β-mercaptoethanol. Samples were loaded and size separated by SDS–polyacrylamide gel electrophoresis and transferred to nitrocellulose membranes (Bio-Rad). Membranes were blocked in 5% blocking buffer (Bio-Rad) in tris-buffered saline with Tween 20 and incubated for 16 hours at 4°C with primary antibodies (all at 1:1000) recognizing these proteins: COPA (SC-398099), COPB1 (SC-393615), COPD (SC-515549), COPE (SC-133195), COPG1 (SC-393977), CDC20 (SC-13162), or ACTB (β-actin; SC-47778) (Santa Cruz Biotechnology); COPZ1 (20440-1-AP), CDK1 (10766-1-AP), or EEA1 (28347-1-AP) (Proteintech); AP2M1 (no. 68196), VPS35 (no. 81453T), PARP (no. 9542), ATF4 (no. 11815), ATF3 (no. 33593), PERK (no. 3192), pRB (S780) (no. 9307), CCNA2 (no. 67955), COL4A1 (no. 75087), CD49A (no. 15574), pTBK1 (no. 5483), or TBK (no. 3504) (Cell Signaling Technology); LC3B (no. AB192890) (Abcam); COPB2 (PA5-96557) (Invitrogen); and COL1A1 (no. A1352) (ABclonal). After incubation with secondary antibodies, signals were detected using a chemiluminescent solution (Kwik Quant) with a Chemi-Doc (Bio-Rad) instrument.

### Immunofluorescence

WI-38 cells were fixed in 3% paraformaldehyde for 10 min and then washed three times with PBS. Cells were permeabilized in 0.25% Triton X-100 for 5 min at 25°C followed by another three washes with PBS. Cells were then incubated with 10% normal goat serum (Invitrogen) for 1 hour at 25°C and then with each primary antibody (diluted in normal goat serum, 1:250) for 16 hours at 4°C; antibodies used recognized GM130 (11308-1-AP), EEA1 (28347-1-AP), and TOM20 (11802-1-AP) from Proteintech; AP2M1 (no. 68196) and VPS35 (no. 81453T) from Cell Signaling Technology; COPB1 (SC-393615) and COPG1 (SC-393977) from Santa Cruz Biotechnology; and LC3B (AB192890) from Abcam. After washing with PBS, cells were incubated with a secondary antibody (1:500) for 1 h at 25°C in the dark. After three additional washes with PBS and incubation for 10 min with DAPI (1:5000), fluorescent signals were analyzed using a Keyence BZ-X710 microscope or a confocal Zeiss LSM 980 Airyscan-2 microscope.

### Transmission electron microscopy (TEM)

For TEM imaging, cultured cells were fixed in 2.5% glutaraldehyde and 3 mM MgCl_2_ in 0.1 M sodium cacodylate buffer (pH 7.2) for 16 hours at 4°C. The buffer was then removed, and the samples were incubated in 2% osmium tetroxide in 0.1 M sodium cacodylate for 1 to 2 hours on ice in the dark. After rinsing in distilled water, the samples were dehydrated in a graded series of ethanol, embedded in Epon (PolySci) resin, and polymerized at 60°C overnight. Sections (60 to 90 nm thick) were cut with a diamond knife on a Leica UCT ultramicrotome and placed on 2 by 1–mm Formvar copper slot grids. They were stained with 3% uranyl acetate (aq.) and lead citrate and then examined using a Hitachi 7600 transmission electron microscope at 80 kV. Images were captured with an AMT CCD XR80 (8-megapixel side mount AMT XR80 high-resolution high-speed camera).

### RNA isolation and RT-qPCR analysis

After homogenization of cell lysates using QIAshredder columns (Qiagen, 79654), total RNA was isolated using the RNeasy Mini kit (Qiagen, 74106), and the concentrations of isolated RNA were measured using a NanoDrop spectrophotometer (Thermo Fisher Scientific). RNA was reverse transcribed using a high-capacity RNA-to-cDNA kit (Thermo Fisher Scientific) following the manufacturer’s instructions. qPCR analysis was performed on a Quant Studio 5 Real-Time PCR System (Thermo Fisher Scientific) using SYBR green master mix (Kapa Biosystems), and the relative mRNA levels were calculated using the 2^−ΔΔCT^ method. Primer sequences are listed in table S5.

### RNA-seq library preparation and sequencing

After checking the quality and quantity of RNA using Agilent RNA Screen Tape on the Agilent TapeStation, 150 ng of high-quality RNA was used to prepare a sequencing library using the Illumina TruSeq Stranded Total RNA Library prep kit (Illumina, no. 20020598). Briefly, after ribosomal RNA depletion and cDNA generation, cDNAs were subjected to 3′ end adenylation, adapter ligation, and purification with AMPure beads (Beckman, no. A63881). The products were size selected with SPRIselect beads (Beckman, no. B23318), enriched by PCR amplification, and purified again with SPRIselect beads to generate the final libraries. The quality and quantity of the sequencing libraries were checked using Agilent DNA 1000 Screen Tape on the Agilent TapeStation. Paired-end sequencing was performed for ∼110 cycles with an Illumina NovaSeq 6000 sequencer with a depth of ∼100 million reads per sample.

### RNA-seq analysis

After sequencing, BCL files were converted to FASTQ files using the bcl2fastq program (version 2.20.0.422). The quality of reads in the FASTQ files was assessed with FastQC tool. The reads were trimmed for adapters using BBDuk from BBTools (version 38.76), mapped to the human reference genome hg38 (Ensembl version 104) using STAR aligner (version 2.7.0f_0328) (*66*), and quantified with featureCounts in the R package Subread (version 2.10.5) (*67*). The differential transcriptomic analysis between samples was performed in the DESeq2 package (version 1.46.0) (*68*) using the Wald test. The transcripts were considered to be differentially abundant if they met statistical significance by the Benjamini-Hochberg adjusted *P* < 0.01. Functional analysis was performed using clusterProfiler (version 4.14.4) (*69*).

### Analysis of mitochondrial membrane potential using JC-1

WI-38 cells were treated with JC-1 (Cell Signaling Technology) following the manufacturer’s protocol. Briefly, cells were incubated in serum-free medium with 10 μM JC-1 for 15 min at 37°C under growth conditions. Cells were then washed twice with 1× PBS, and the fluorescence intensity of JC-1 aggregates (red) and JC-1 monomers (green) was analyzed with a Keyence BZ-X710 fluorescence microscope.

### Measurement of mitochondrial respiration

For mitochondrial respiration, senescence induction and transfections were performed in Seahorse 24-well Cell Culture plates, and the assays were run on the Seahorse XFe24 Flux Analyzer (Agilent Technologies). One well per plate was left blank for background correction, and ports were preloaded in the Seahorse 24-well Flux Assay plates [port A: oligomycin; port B: carbonyl cyanide *p*-trifluoromethoxyphenylhydrazone (FCCP); port C: antimycin A and rotenone]. Final concentrations in the wells after injection were 1.0 μM oligomycin, 3.0 μM FCCP, and 0.5 μM antimycin A/rotenone. Before loading the cell culture plate in the Flux Analyzer, the culture media were removed and replaced with 525 μl of Agilent Base Media supplemented with 10 mM glucose, 1.0 mM sodium pyruvate, and 2 mM glutamine that had been warmed to 37°C and pH adjusted to 7.4. The cell culture plate was then incubated for 1 hour at 37°C before being read on the Flux Analyzer. The measurement cycles were as follows: baseline, three cycles with a 3-min mix, 2-min wait, and 3-min measure; port A oligomycin injection, two cycles with a 3-min mix, 2-min wait, and 3-min measure; port B FCCP injection, two cycles with a 3-min mix, 2-min wait, and 3-min measure; port C antimycin A/rotenone injection, two cycles with a 3-min mix, 2-min wait, and 3-min measure. The OCR and ECAR (extracellular acidification rate) were analyzed using Agilent Wave 2.6.4 software.

### Bio-Plex analysis of cytokine secretion

For the multiplex measurement of secreted cytokines, the medium from cultured cells was collected and centrifuged at 16,000*g* for 4 min at 4°C. Custom human Luminex assay kits were designed (Biotechne) to include IL-6, IL-8, and CXCL2. Standards, blanks, and the undiluted supernatant were incubated with the microparticle cocktail solution for 2 hours at 25°C, with the biotin antibody cocktail for 1 hour, and with streptavidin-PE for 30 min, all with rotation. After washes and additional incubation at 850 rpm for 2 min, the plate was analyzed on a Bio-Plex 200 instrument (Bio-Rad) with the following settings: sample volume of 50 μl, Bio-Plex Magplex beads (magnetic), double discriminator gates set at 8000 and 23,000, low rp1 target cal for the cal1 settings, and 50 counts per region.

### Caspase 3/7 Glo assay

The activity of caspases 3 and 7 was measured in a 96-well solid white-bottom plate following the manufacturer’s instructions (Promega, G8091). The caspase 3/7 substrate, dissolved in the provided buffer solution, was added to the media of each well. Luminescence was measured using the Promega GloMax Explorer microplate reader every 30 min for 90 min, and peak reading was used for analysis.

### MS-based proteomics of immunoprecipitated proteins

Immunoprecipitated samples were processed for in-solution trypsin/LysC digestion and analyzed by liquid chromatography–tandem MS (LC-MS/MS) (Poochon Scientific). The resulting MS data were searched against the human protein sequence database using Proteome Discoverer 2.5 (Thermo Fisher Scientific). The abundance of identified proteins was analyzed using a workflow established in the R package DEP, version 1.28.0 (*70*). Only proteins detected in at least two of three replicates in COPI IP material were included for analysis. Proteins showing enrichments of log_2_FC > 5 (*P*_adj_ < 0.05) in COPI IP compared to IgG IP were deemed associated with the COPI complex.

### Immunoprecipitation

Cells were lysed in radioimmunoprecipitation assay buffer (10 mM tris-HCl, pH 7.4, 1% Nonidet P-40, 1 mM EDTA, 0.1% SDS, and 150 mM NaCl) supplemented with a protease/phosphatase inhibitor (Thermo Fisher Scientific), incubated on ice for 10 min, and centrifuged for 20 min at 13,200 rpm at 6°C. The supernatant was collected, and 1 mg of protein lysate was incubated with 10 μg of antibody (COPB1: 27469-1-AP; COPE: 11457-1-AP; COPZ1: 20440-1-AP; all from Protein Tech) or Normal Rabbit IgG (Cell Signaling Technology, no. 2729S) and 50 μl of Dynabeads protein G (Thermo Fisher Scientific, no. 10004D). After overnight incubation at 4°C on a spinning rotor, the beads were washed five times with radioimmunoprecipitation assay buffer. The complexes were dissociated from the beads in 2× Laemmli sample buffer containing 10% β-mercaptoethanol.

### Statistical analysis and graphs

Bands on Western blots were quantified by densitometry analysis using ImageJ. The graphs and statistical analysis were prepared using Prism GraphPad 10. All measurable data were represented as the means ± standard error of the mean (SEM). Statistical significance was assessed by unpaired Student’s *t* test for comparisons between two groups. An analysis of variance (ANOVA) followed by Dunnett’s post hoc test was performed for comparisons involving more than two groups, each with a control group.
