# Supplementary material for: Distinct roles of COPI proteins attenuated in cell senescence
Source: Sci Adv. 2026 Jul 23;12(30):eaec2786. doi: 10.1126/sciadv.aec2786 (PMC13394425; doi:10.1126/sciadv.aec2786)
Supplement: Supplementary file 1 — Figs. S1 to S9 Tables S2 and S5 Legends for tables S1, S3 and S4 [file sciadv.aec2786_sm.pdf]

Science Advances
AAAS

# Supplementary Materials for

## Distinct roles of COPI proteins attenuated in cell senescence

Krystyna Mazan-Mamczarz *et al.*

Corresponding author: Krystyna Mazan-Mamczarz, krystyna.mazan-mamczarz@nih.gov; Myriam Gorospe, myriam-gorospe@nih.gov

*Sci. Adv.* **12**, eaec2786 (2026)
DOI: 10.1126/sciadv.aec2786

**The PDF file includes:**

Figs. S1 to S9
Tables S2 and S5
Legends for tables S1, S3 and S4

**Other Supplementary Material for this manuscript includes the following:**

Tables S1, S3 and S4

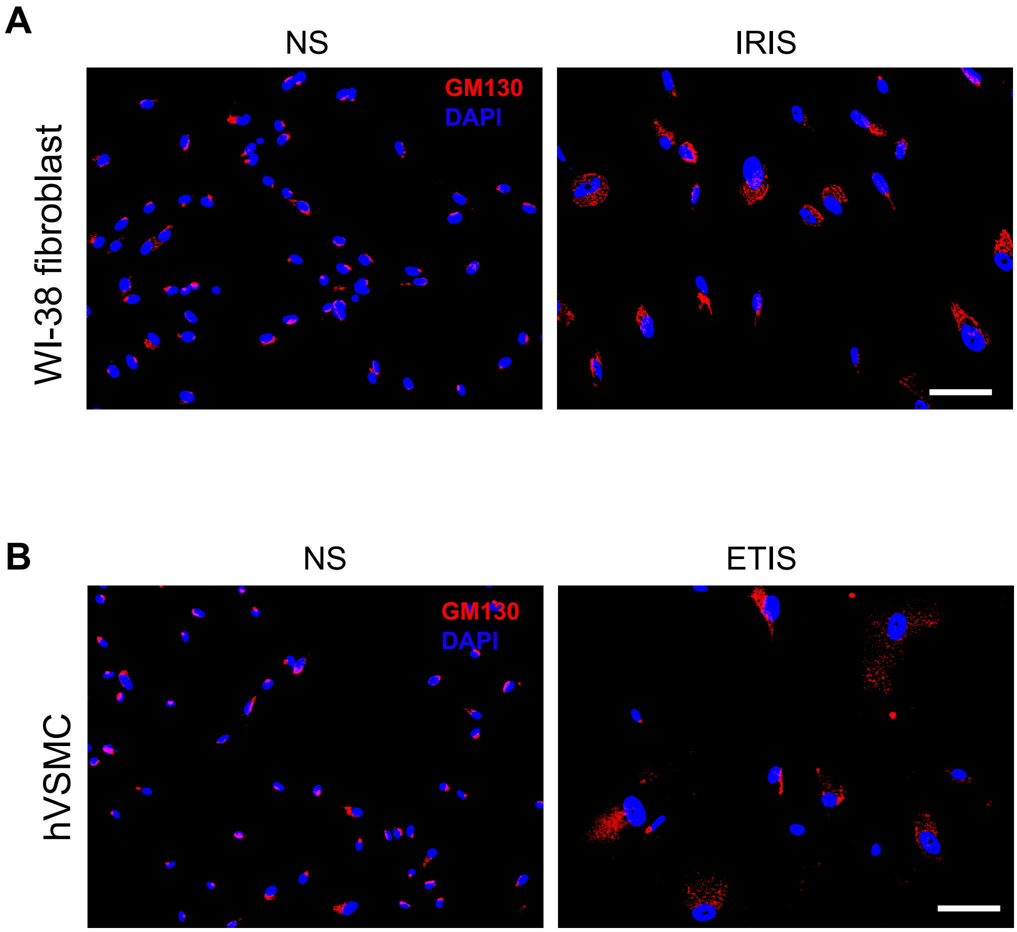


**Fig. S1. Visualization of changes in the Golgi compartment by immunofluorescence microscopy.** The Golgi compartment was visualized by immunofluorescence staining with an antibody recognizing Golgi membrane protein GM130 (red) and DAPI was used to stain the cell nuclei (blue). Scale bar, 100 µm. **(A)** WI-38 fibroblasts that were non-senescent (NS) or were subjected to irradiation-induced senescent (IRIS) and analyzed 10 days later. **(B)** NS and etoposide-induced (ETIS) senescent human vascular smooth muscle cells (hVSMC).

**A**

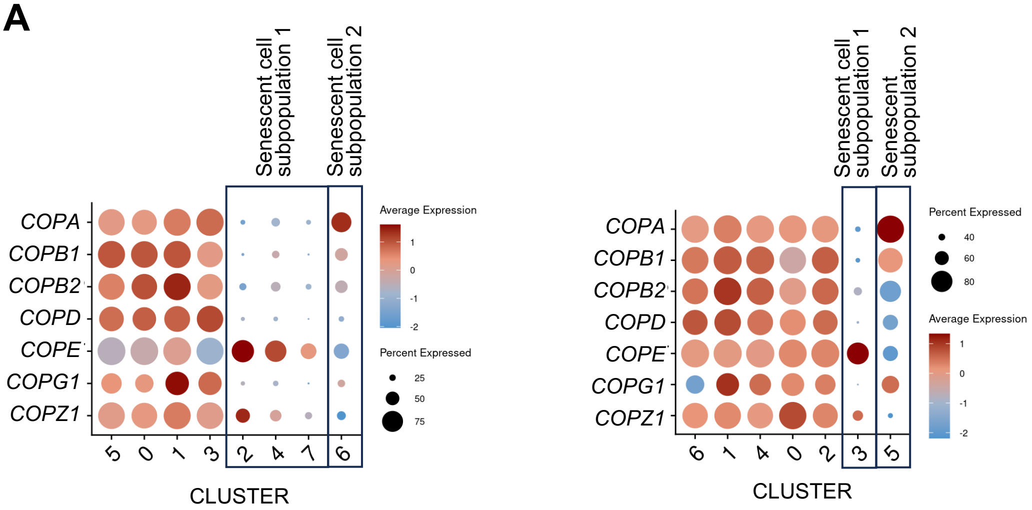


**B**

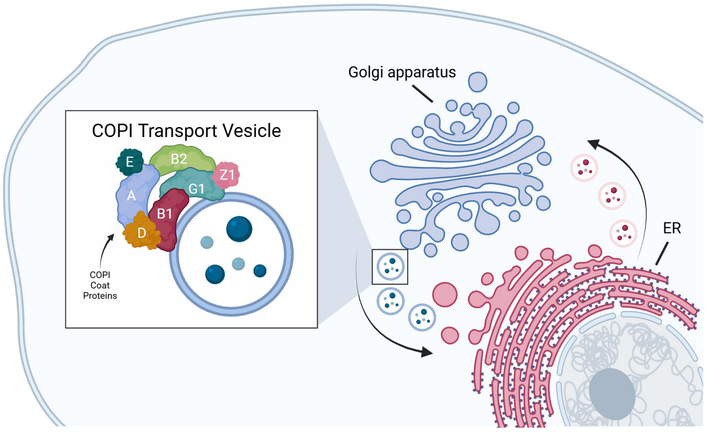


**Fig. S2. Expression of COPI subunits in senescent cells.** (**A**) Levels of mRNAs encoding COPI components in two different subpopulations of senescent cells that were clustered by scRNA-seq analysis in Wechter *et al*. (2023), in different models of cell senescence (*left*) and a time-course of etoposide-induced senescence (*right*). Dot color indicates scaled average expression and dot size shows the percentage of cells expressing specific mRNAs in each condition. '*Senescent cell subpopulation 1*' comprised cells that expressed traditional senescence markers such as *p16* (*CDKN2A*) mRNA, and '*Senescent cell subpopulation 2*' comprised cells that expressed long noncoding RNAs and in which splicing was dysregulated (Wechter et al. 2023). (**B**) Schematic of COPI coat proteins on vesicles being transported between the Golgi apparatus and the endoplasmic reticulum (ER).

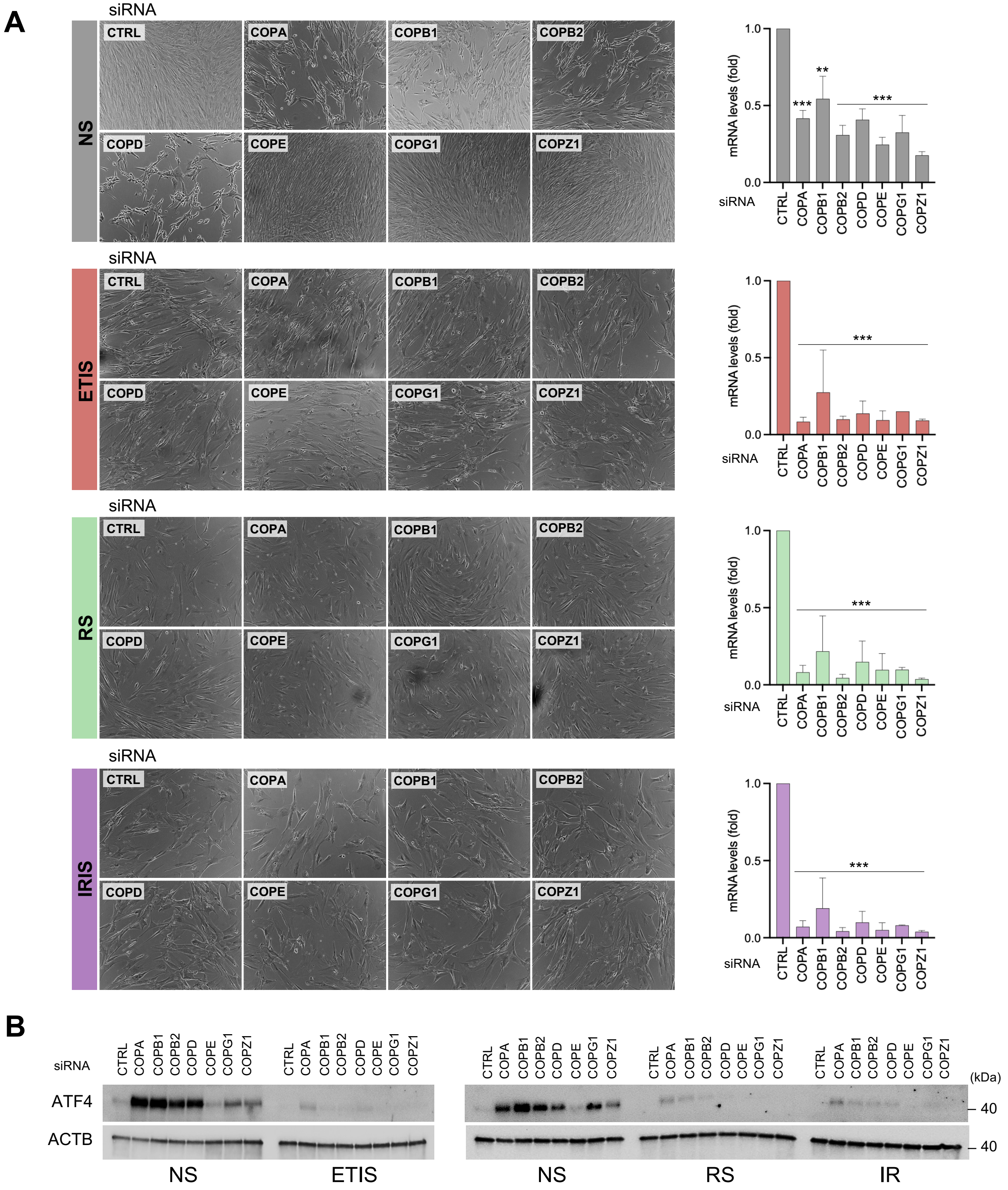


**Fig. S3. Analysis of the impact of silencing each COPI subunit in different WI-38 senescence models.** WI-38 fibroblasts were either proliferating (NS), treated for 10 d with 50 µM of etoposide (ETIS), cultured until replicative exhaustion at PDL 54 (RS), or exposed to 10 Gy of ionizing radiation and cultured for an additional 10 days (IRIS). Cells were then transfected with either non-targeting siRNA (CTRL) or siRNAs to silence each subunit of the COPI complex, 72 h before collection. **(A)** Representative phase-contrast micrographs depicting the appearance of each transfected population (*left*), RT-qPCR analysis was used to assess transfection efficiency by evaluating the levels of individual COPI mRNA in the group (*right*); the relative mRNA levels were normalized to *GAPDH* mRNA levels and compared to NS CTRL siRNA cells. Statistical significance in was assessed by ANOVA followed by post hoc Dunnett's test (**, $p < 0.01$; ***, $p < 0.001$) relative to CTRL siRNA in each group (n=2). **(B)** Western blot analysis of ATF4 protein levels in the the samples from (A); ACTB, loading control.

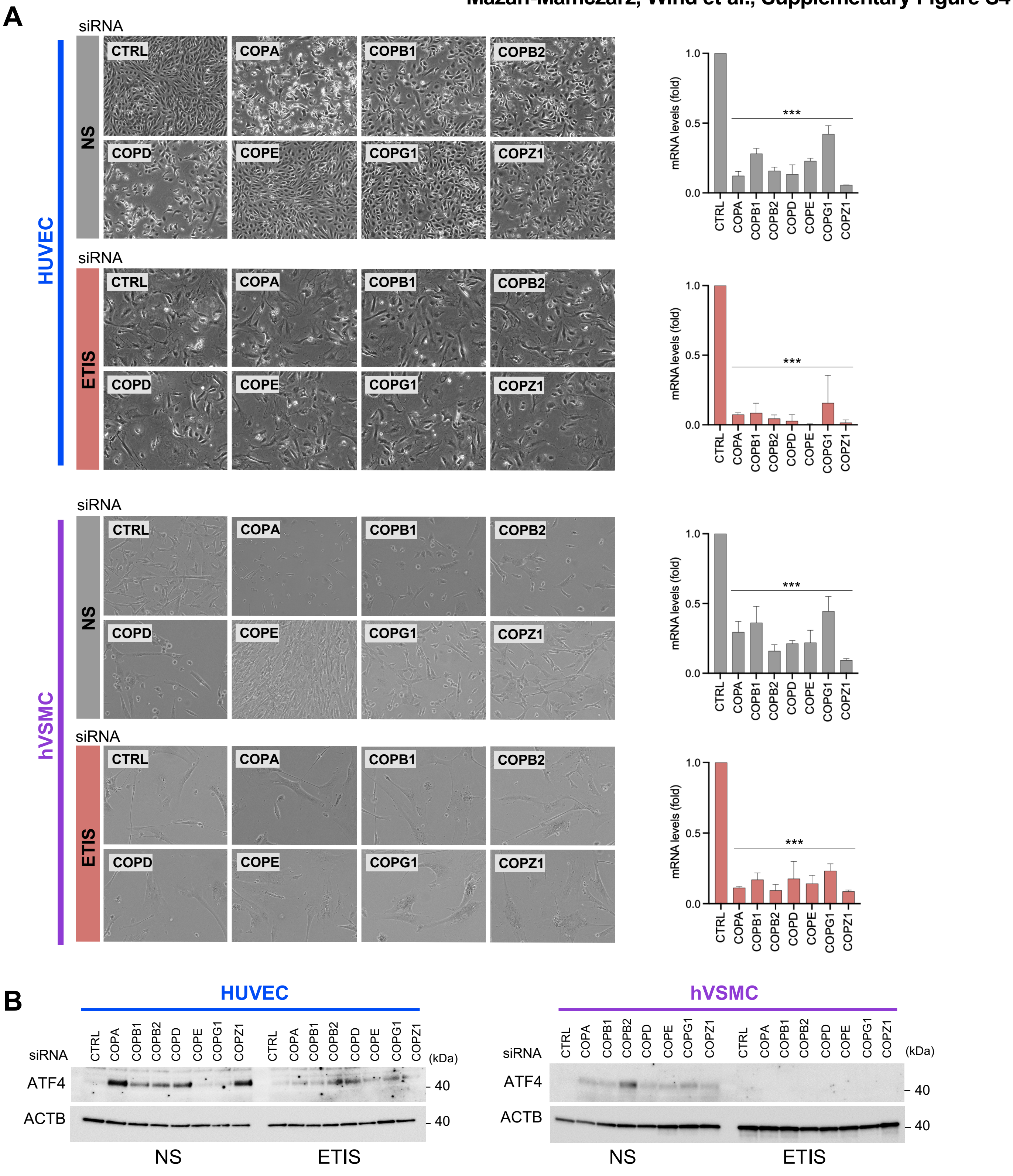


**Fig. S4. Analysis of the impact of silencing each COPI subunit in human umbilical vein endothelial cells (HUVECs) and human vascular smooth muscle cells (hVSMC).** HUVECs were either NS or cultured in media with 10 µM of etoposide for 3 d and cultured for an additional 9 days (ETIS). hVSMCs were either NS or cultured in media with 50 µM of etoposide for 10 d (ETIS). **(A)** Representative phase-contrast micrographs depicting the appearance of each transfected population (*left*), RT-qPCR analysis was used to assess transfection efficiency by evaluating the levels of each individual COPI mRNA subunit (*right*); the relative mRNA levels were normalized to *GAPDH* mRNA levels and compared to NS CTRL siRNA cells. Statistical significance was assessed by ANOVA followed by Dunnett's post hoc test (***, $p < 0.001$) relative to CTRL siRNA in each group (n=3). **(B)** Western blot analysis of ATF4 protein levels in the samples from (A); ACTB, loading control.

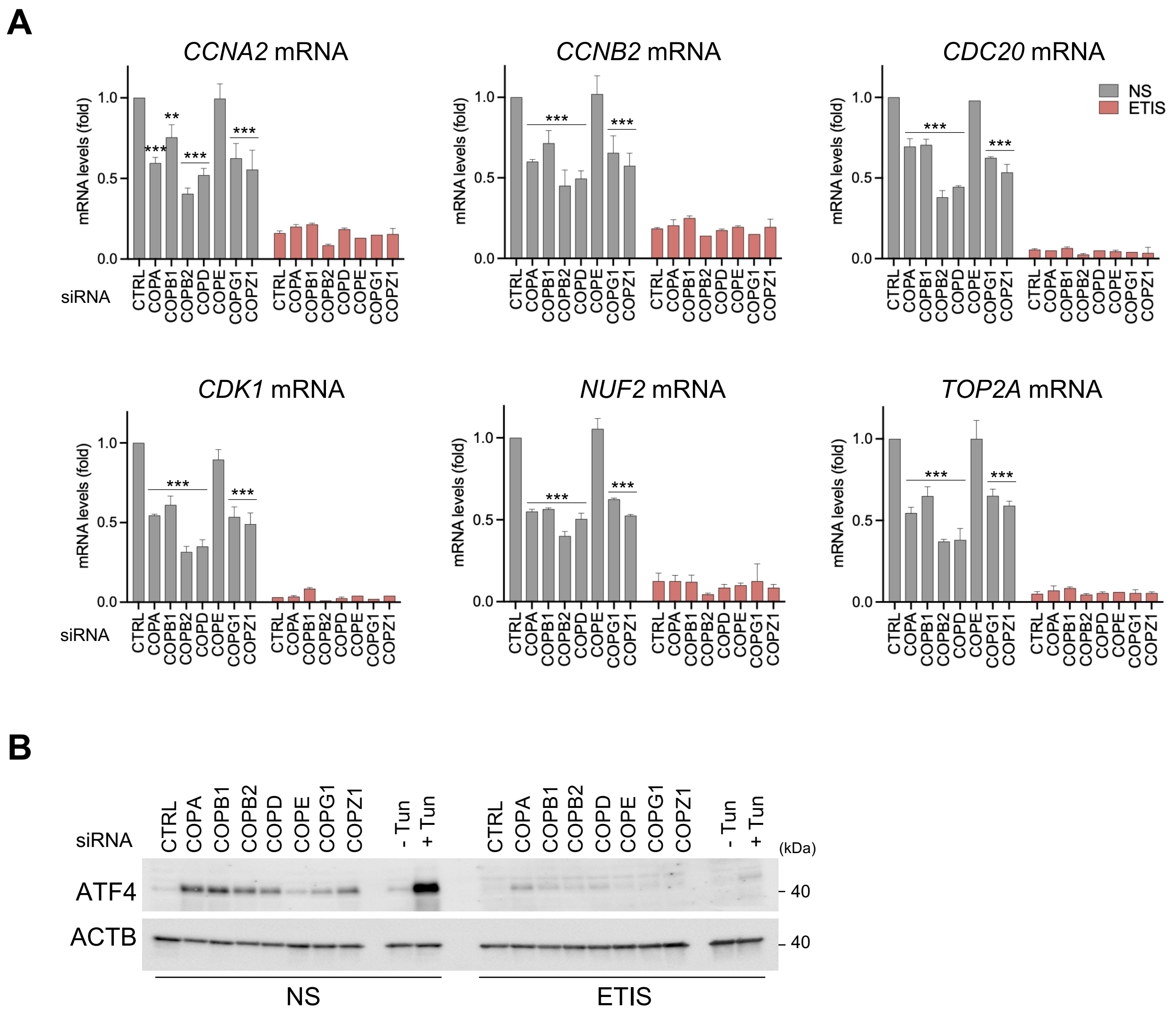


**Fig. S5. Extended analysis of functional pathways affected by silencing each COPI subunit in NS and ETIS WI-38 cells. (A)** RT-qPCR validation of the mRNAs identified by RNA-seq analysis (main Figure 3, D and E) as encoding proteins involved in DNA replication and cell division (Fig. 3 D and E): *CCNA2*, *CCNB2*, *CDC20*, *CDK1*, *NUF2*, and *TOP2A* mRNAs (n=3). Relative mRNA levels were normalized to *GAPDH* mRNA levels and compared to NS CTRL siRNA cells. The statistical significance was assessed by ANOVA followed by Dunnett's post hoc test (*, $p < 0.05$; **, $p < 0.01$; ***, $p < 0.001$) in comparison to CTRL siRNA in each group (n=2). **(B)** Western blot validation of the abundance of ATF4 protein after silencing each of the COPI subunits, and in cells treated with tunicamycin (Tun, 250 ng/ml, 4 h; Sigma).

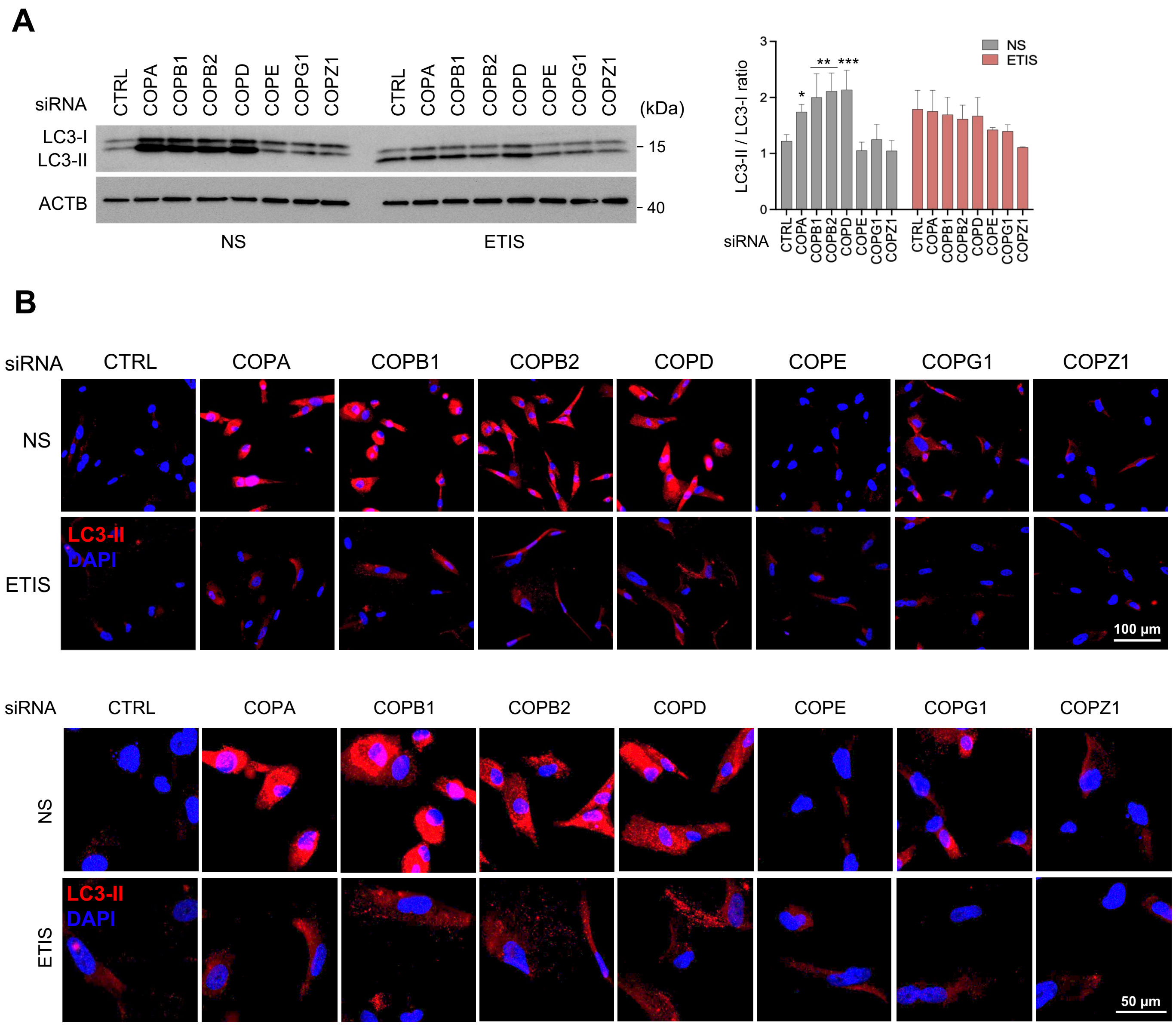


**Fig. S6. Impact of silencing each COPI subunit on autophagy in NS and ETIS WI-38 cells.** WI-38 cells were processed as described in Figure 4A. (**A**) The levels of LC3 proteins were assessed by western blot analysis; ACTB was included as a loading control (*left*). The relative intensities of quantified LC3 proteins levels were represented as LC3-II/LC3-I ratios (*right*). The statistical significance was assessed by ANOVA followed by Dunnett's post hoc test (*, $p < 0.05$; **, $p < 0.01$; ***, $p < 0.001$; ****, $p < 0.0001$) relative to CTRL siRNA in each group (n=3). (**B**) Representative immunofluorescent micrographs of LC3-II-positive autophagic foci (red); nuclei (blue) were labeled with DAPI.

**A**

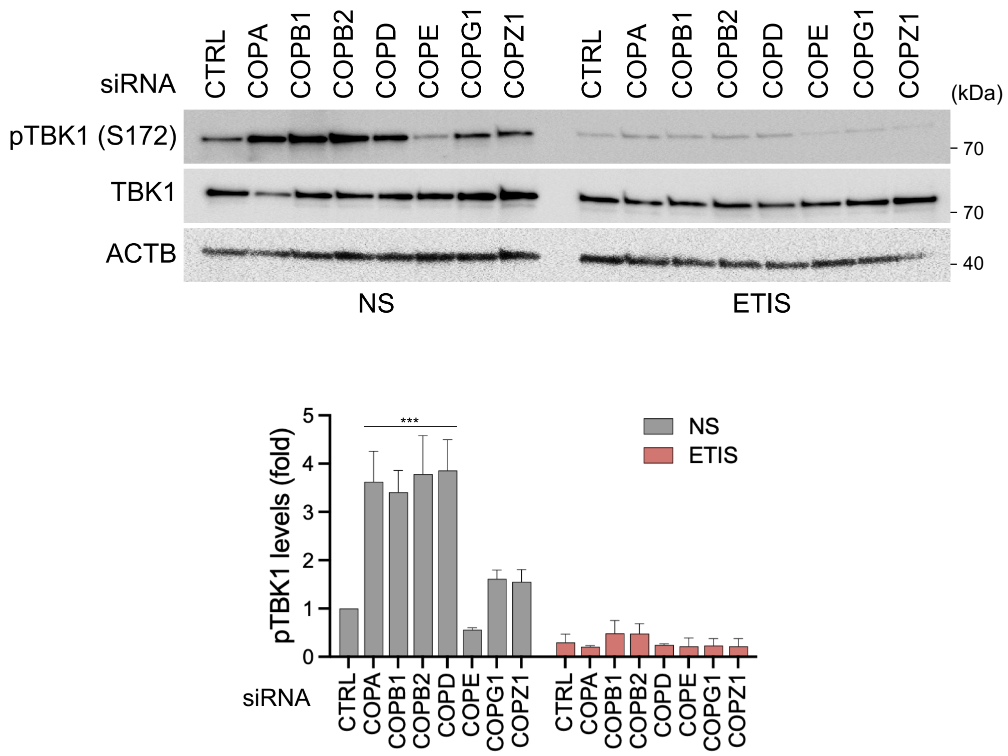


**B**

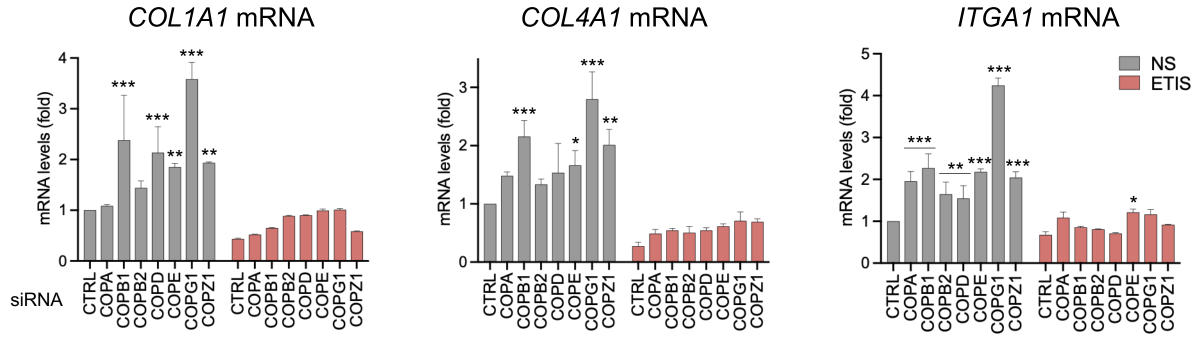


**Fig. S7. Effect of silencing each COPI subunit on STING signaling and expression of ECM factors in WI-38 cells.** WI-38 cells were processed as described in Figure 4A. (**A**) The abundance of pTBK1 and TBK1 was assessed by western blot analysis (n=2). Representative images (*top*) and pTBK1 quantification (*bottom*). pTBK1 levels were normalized to ACTB levels and compared to CTRL siRNA. **(B)** The levels of mRNAs encoding extracellular matrix proteins were assessed by RT-qPCR analysis (n=3). In (A, B), statistical significance was determined by ANOVA followed by Dunnett's post hoc test in comparison to the CTRL siRNA in each group (*, $p < 0.05$; **, $p < 0.01$; ***, $p < 0.001$).

**A**

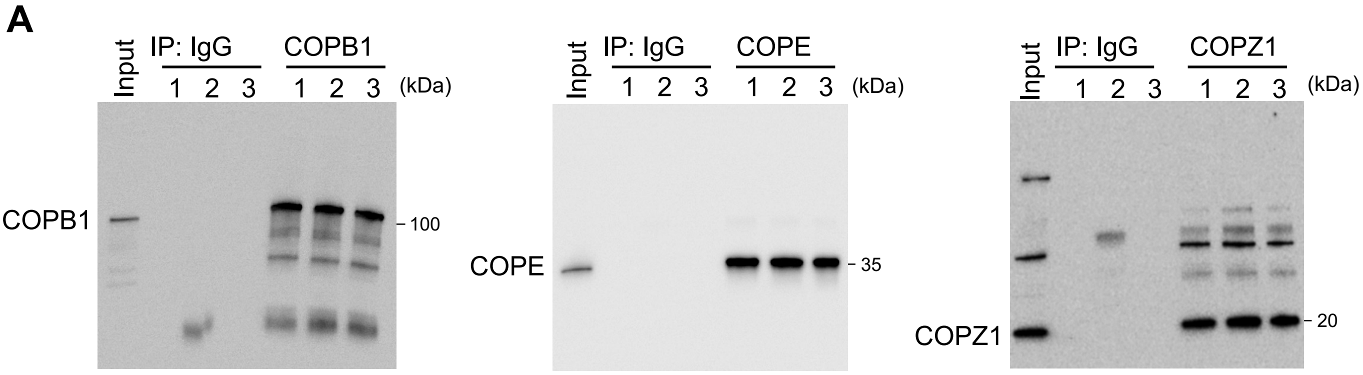


**B**

**Golgi Membrane**

| | | |
|---|---|---|
| ARF3 | COPB2 | RAB1B |
| ARL1 | COPD | RAB2A |
| ASAP1 | COPE | RAB6A |
| CAV2 | COPG1 | SEC16A |
| CDC42 | COPG2 | SEC22B |
| COG4 | COPZ1 | SEC23A |
| COPA | COPZ2 | SURF4 |
| COPB1 | KDELR2 | TAPBP |
| | | VAPA |

**Golgi Membrane Endosome**

| | | |
|---|---|---|
| AP3D1 | DBNL | RAB10 |
| ATP6AP2 | IGF2R | RAB14 |
| | PRKCI | RAB21 |

**Endosome**

| | |
|---|---|
| ANXA1 | EZR |
| AP2A1 | FKBP15 |
| AP2A2 | FLOT1 |
| AP2B1 | GAPVD1 |
| AP2M1 | GRIPAP1 |
| AP2S1 | HGS |
| AP3M1 | MON2 |
| ARF6 | RAB13 |
| CHMP1A | RAB3B |
| CHMP1B | RAC1 |
| CHMP4B | SNX18 |
| CHMP6 | TF |
| DIAPH3 | VPS18 |
| EEA1 | VPS25 |
| EHD1 | VPS35 |
| EHD2 | VPS35L |
| ENTR1 | VPS36 |

**Other**

| | | |
|---|---|---|
| ALOX15 | HEATR5B | SEPTIN2 |
| ANK3 | HYOU1 | SEPTIN5 |
| ATP2A2 | LILRB4 | SEPTIN8 |
| ATXN2 | LRP1B | SERPINE1 |
| BTBD9 | MIA3 | SH3BP4 |
| C17orf75 | MIB1 | SH3KBP1 |
| C3 | PCLO | SNX9 |
| C4A | PDLIM7 | SPG11 |
| CBLL1 | PFN2 | TBC1D10B |
| CNN2 | RAP1B | TBC1D10C |
| CSNK1D | RP2 | TFG |
| DCTN1 | S100A10 | TGM2 |
| DNM1 | SDC4 | VAV3 |
| DPY30 | SDCBP | WASL |
| FMR1 | SEC13 | YES1 |
| GREM1 | SEC31A | YIPF5 |

**C**

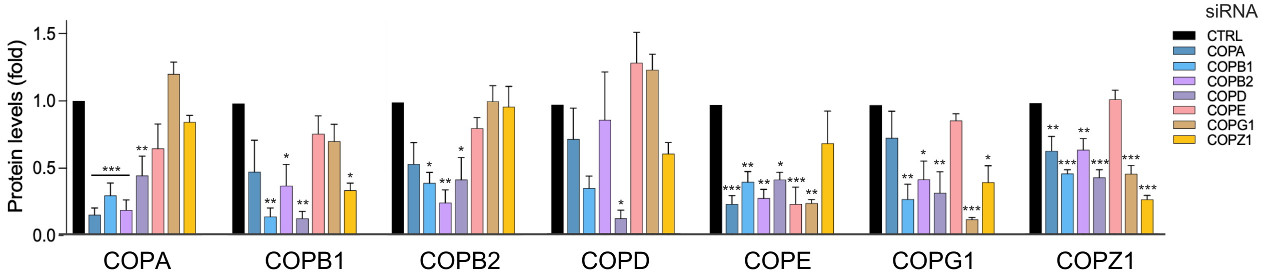


**Fig. S8. Interaction of COPI complex proteins.** **(A)** Western blot analysis of COPB1, COPE, and COPZ1 to assess the efficiency of immunoprecipitation (IP) with the same antibodies, compared to control IgG IP; the three repeats were used for mass spectrometry (MS) proteomic analysis. **(B)** Components of the GO BP dataset 'Vesicle Mediated Transport' that were significantly enriched in MS data following IP with antibodies recognizing COPB1, COPE, or COPZ1. **(C)** Quantification of COPI proteins abundance after individually silencing each subunit of the COPI complex represented in Fig. 7B. Protein levels were normalized to ACTB levels and compared to CTRL siRNA. Statistical significance was determined using ANOVA followed by Dunnett's post hoc test relative to CTRL siRNA cells (n=3) (*, $p < 0.05$; **, $p < 0.01$; ***, $p < 0.001$).

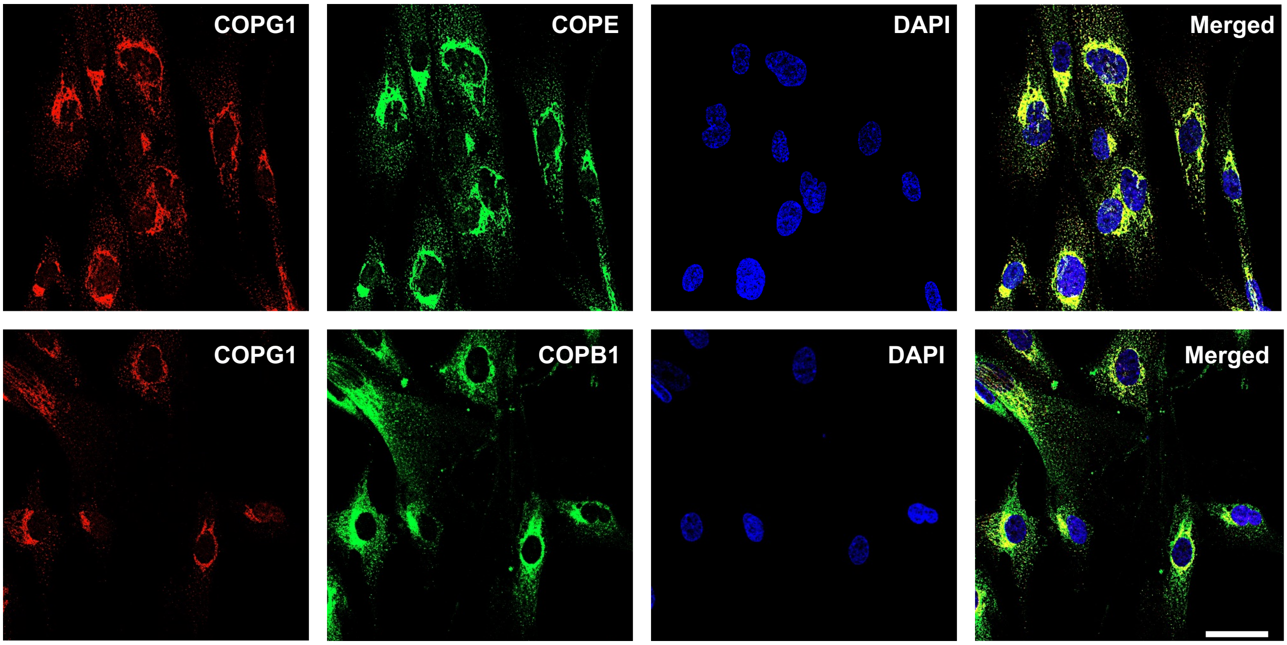


**Fig. S9. Colocalization of COPI components.** Representative z-stack confocal immunofluorescence micrographs to visualize the colocalization of COPB1 and COPE with COPG1. Scale bar, 40 μm.

| siRNA | Source |
| --- | --- |
| ON-TARGETplus Human COPA siRNA | Dharmacon Cat # J-011835-05 |
| ON-TARGETplus Human COPB1 siRNA | Dharmacon Cat # J-017940-09 |
| ON-TARGETplus Human COPB2 siRNA | Dharmacon Cat # J-019847-08 |
| ON-TARGETplus Human ARCN1/COPD siRNA | Dharmacon Cat # J-013063-09 |
| ON-TARGETplus Human COPE siRNA | Dharmacon Cat # J-017632-11 |
| ON-TARGETplus Human COPG1 siRNA | Dharmacon Cat # J-019138-07 |
| ON-TARGETplus Human COPZ1 siRNA | Dharmacon Cat # J-020293-09 |
| *Silencer*™ Select Negative Control No. 2 siRNA | Invitrogen Cat # 4390846 |

**Table S2. List of siRNAs**. Sequences of siRNAs employed in transfection experiments to silence each COPI subunit.

| Transcript Name | Primer | Oligo Sequence |
|---|---|---|
| *COPA* | Forward | GCTACACTATGTCAAGGACCG |
| *COPA* | Reverse | TGAATACTGGAAACTTGGAACCA |
| *COPB1* | Forward | CTTCCTGGACTTCTGATGACC |
| *COPB1* | Reverse | TGCATCACATACAAGGATCATCT |
| *COPB2* | Forward | GAGCGGATGACATGCAGATT |
| *COPB2* | Reverse | TCCCAGTCCCAGAGCTTAAT |
| *ARCN1/COPD* | Forward | TGTTCAGAGCCGTCAGAGAG |
| *ARCN1/COPD* | Reverse | TGCCTCCAGATACTGCAGAG |
| *COPE* | Forward | TCTACATCGGCAGCTACCAG |
| *COPE* | Reverse | CGAGGAGGGCTTGATCTCAT |
| *COPG1* | Forward | TGGTCTGACTGTGTCCATCC |
| *COPG1* | Reverse | GTTTGACTGCTGTGATGGGG |
| *COPZ1* | Forward | GGAAGGCCTGACAGTGGTAT |
| *COPZ1* | Reverse | GCAGTGCTCGCTTTTCTACA |
| *CDKN2A* | Forward | CGATGTCGCACGGTACCT |
| *CDKN2A* | Reverse | TCTGGTTCTTTCAATCGGGGA |
| *CDKN1A* | Forward | AGTCAGTTCCTTGTGGAGCC |
| *CDKN1A* | Reverse | CATGGGTTCTGACGGACAT |
| *DPP4* | Forward | CGTGGAAGGTTCTTCTGGGA |
| *DPP4* | Reverse | TTTGCGACTGTCAGCTGTAG |
| *IL6* | Forward | TGTGAAAGCAGCAAAGAGGC |
| *IL6* | Reverse | ACCAGGCAAGTCTCCTCATT |
| *IL8* | Forward | GAGACTTGCCTGGTGAAAATCA |
| *IL8* | Reverse | CTCTGGCTTGTTCCTCACTAC |
| *TNF* | Forward | CCTCAGCCTCTTCTCCTTCC |
| *TNF* | Reverse | TTAGAGAGAGGTCCCTGGGG |
| *IFNB1* | Forward | ACGCCGCATTGACCATCTAT |
| *IFNB1* | Reverse | GTCTCATTCCAGCCAGTGC |
| *GDF15* | Forward | AGTTGCACTCCGAAGACTCC |
| *GDF15* | Reverse | GGTGTTCGAATCTTCCCAGC |
| *CCNA2* | Forward | CCAGGAGAATATCAACCCGGA |
| *CCNA2* | Reverse | GGAACGGTGACATGCTCATC |
| *CCNB2* | Forward | AGTTCCAGTTCAACCCACCA |
| *CCNB2* | Reverse | GCAGAGCAAGGCATCAGAAA |
| *CDC20* | Forward | ATCCTTCGGCTCAGTGGAAA |
| *CDC20* | Reverse | AGGGAAGGAATGTAACGGCA |
| *CDK1* | Forward | AGCCTAGCATCCCATGTCAA |

| | | |
|---|---|---|
| *CDK1* | Reverse | TCAGTGCCATTTTGCCAGAA |
| *NUF2* | Forward | CCTGCCTTCATGTCAGTTGG |
| *NUF2* | Reverse | GTCCTCCAAGTTCAGGCTCT |
| *TOP2A* | Forward | ACAGGTGGTCGAAATGGCTA |
| *TOP2A* | Reverse | CATCTCACCAGCTCTTCCCA |
| *COL1A1* | Forward | ATGACCGAGACGTGTGGAAA |
| *COL1A1* | Reverse | GCAGTTCTTGGTCTCGTCAC |
| *COL4A1* | Forward | GCAAACCCGGAAAAGATGGT |
| *COL4A1* | Reverse | AGGACCTGCTTCACCCTTTT |
| *ITGA1* | Forward | GGTTCACGGACACCTTCAAC |
| *ITGA1* | Reverse | CCACGACCAGCCACTTATTG |
| *GAPDH* | Forward | CTCTGCTCCTCCTGTTCGAC |
| *GAPDH* | Reverse | ACGACCAAATCCGTTGACTC |

**Table S5. List of qPCR oligomer sequences.** Sequences of oligomer pairs used for mRNA detection in RT-qPCR analyses conducted in the study.

## LEGENDS FOR SUPPLEMENTARY EXCEL AND TEXT FILES

**Table S1. RNA interference (RNAi) screen for EV uptake.** A high-throughput screen was performed using the whole-genome Silencer Select Human Genome siRNA Library V4 (Ambion). Table includes the results for all screened genes (on-target-gene), the individual results of three independent siRNAs per gene (on-target-siRNA), and off-target gene contribution (off-target-gene). The changes in EV uptake were evaluated using the median seed-corrected Z-score (exosome.z.gespeRcorrected.median).

**Table S3: Differentially abundant transcriptomes after silencing each COPI subunit.** Transcriptomes after silencing each COPI subunit were evaluated by RNA-sequencing (RNA-seq) analysis using the DESeq2 R package. Cells transfected with siRNAs targeting individual COPI components were compared to cells transfected with Control siRNA (CTRLsiRNA), and the differences in abundance of each transcript were assessed by $\log_2$ Fold Change (log2FoldChange).

**Table S4. Proteins identified by immunoprecipitation of COPI subunits followed by mass spectrometry (IP-MS) analysis.** Whole-cell lysates from WI-38 cells were immunoprecipitated using antibodies that recognized COPB1, COPE, COPZ1, or IgG, followed by mass spectrometry (MS)-based proteomic analysis. Table indicates the abundance of the proteins identified by IP-MS in each immunoprecipitated sample.
